# The formation of preference in risky choice

**DOI:** 10.1371/journal.pcbi.1007201

**Published:** 2019-08-29

**Authors:** Moshe Glickman, Orian Sharoni, Dino J. Levy, Ernst Niebur, Veit Stuphorn, Marius Usher

**Affiliations:** 1 The School of Psychological Sciences, Tel Aviv University, Tel Aviv, Israel; 2 Coller School of Management, Tel Aviv University, Tel Aviv, Israel; 3 Sagol School of Neuroscience, Tel Aviv University, Tel Aviv, Israel; 4 Department of Neuroscience and Krieger Mind/Brain Institute, Johns Hopkins University, Baltimore, Maryland, United States of America; Oxford University, UNITED KINGDOM

## Abstract

A key question in decision-making is how people integrate amounts and probabilities to form preferences between risky alternatives. Here we rely on the general principle of integration-to-boundary to develop several biologically plausible process models of risky-choice, which account for both choices and response-times. These models allowed us to contrast two influential competing theories: i) within-alternative evaluations, based on multiplicative interaction between amounts and probabilities, ii) within-attribute comparisons across alternatives. To constrain the preference formation process, we monitored eye-fixations during decisions between pairs of simple lotteries, designed to systematically span the decision-space. The behavioral results indicate that the participants' eye-scanning patterns were associated with risk-preferences and expected-value maximization. Crucially, model comparisons showed that within-alternative process models decisively outperformed within-attribute ones, in accounting for choices and response-times. These findings elucidate the psychological processes underlying preference formation when making risky-choices, and suggest that compensatory, within-alternative integration is an adaptive mechanism employed in human decision-making.

## Author summary

Decision-making under risk requires a selection between alternatives, such as lotteries, which offer a reward with a specified probability. Human decision between such alternatives is at the center of the normative decision theory, which assumes that decisions are rationally made by forming a value for each alternative and selecting the alternative with the highest value. To this day, there is still a considerable debate on how such values are computed. While the normative theory assumes that values of the alternatives reflect the statistically expected rewards, more recent theories have argued that alternative-values are not computed, and choices are only based on sequentially comparing the alternatives on amounts or on probabilities. Here, we carried out an experimental investigation of risky decision-making, in which participants chose between pairs of simple lottery alternatives that systematically span a range of probabilities and amounts, while we tracked their eye positions during the decision-making process. We found that the participants are sensitive to the expected-utility of the alternatives, as predicted by the normative decision theories, but they also exhibit risk-biases that correlate with the eye-scanning patterns. We then carry out computational modeling, comparing preference-formation models on their ability to account for both choices and their reaction-times. The results provide strong support for normative models, which assume that the values of the alternative are computed via a multiplicative function of the amounts and probabilities. These results suggest that humans are closer to normative principles than previously assumed, and motivate further investigation into the neural mechanism that mediates these multiplicative computations.

## Introduction

Decision-making under risk is ubiquitous in daily activities, such as deciding whether to take an umbrella when the weather forecast predicts 50% chance for rain, or whether to purchase a lottery ticket with a winning probability of 1%. Such decisions are difficult because the outcomes of the alternatives are only known with some probability, and thus they are subject to risk tradeoffs. For example, when deciding between a lottery that offers $100 with a probability of 50% and an offer of $40 with certainty, one needs to balance between the appeal of the attractive amount ($100) and the risk of getting nothing (rather than gaining $40 for certain). Choices between such lotteries were the subject of intensive research in economics and experimental psychology that investigated how humans make risky decisions, starting from the normative Expected-Utility (*EU;* [1]), followed by random utility models [2] and culminating with Cumulative Prospect Theory (*CPT*; [3–6], see also Transfer of Attention eXchange [*TAX*], for a related type of model [7]). Yet despite the impressive success of *CPT* in accounting for risky choice data (e.g., the dependence of risk-aversion on the magnitude of the outcomes' probabilities [8]), the theory has been criticized for making assumptions that are inconsistent with capacity limitations of human online information processing, and for not explicating the process by which the preferences are formed [9,10].

Several process theories were developed to account for risky choice. First, heuristic models, such as Priority Heuristic (*PH*), suggest that preferences are not formed via a compensatory process of averaging over all outcomes (like in *EU* and *CPT*), but rather via a sequential process of comparing the alternatives over one specific attribute (probability or amount) at a time, in a specified order, and stopping at the first instance in which a termination criterion is satisfied [9]. Second, a number of models have relied on the sequential-sampling framework [11–14], which successfully accounted for choices in perceptual tasks, in order to develop a process model of risky choice. For example, in Decision Field Theory (*DFT*; [15]), as attention fluctuates between the alternatives, the preference dynamically evolves by integrating amounts, which are sampled with a frequency that is associated with their (subjective) probabilities [16]. In the Decision by Sampling model (*DbS*; [17–19]), like in *PH*, the sampling involves comparisons between the values of the alternatives on a specific attribute (i.e., amounts or probabilities, but not both). However, unlike *PH*, *DbS* does not assume a fixed order of attribute sampling, nor that the decision is settled at a single comparison, but rather a stochastic sampling, which continues until the accumulated difference of favorable comparisons reaches a decision boundary. Critically, as opposed to *EU* or *CPT*, in *DbS* the processing takes place within-attributes (i.e., comparison between amounts or between probabilities). Finally, in the Parallel Constraint Satisfaction model (*PCS*; [20]), a compensatory within-alternative process similar to *EU* (i.e., multiplication of amounts and probabilities) is carried out in a parallel and automatic manner; this process is mediated by a connectionist network of bottom-up and top-down connections. Although several qualitative predictions of the *PCS* model have been confirmed [20], this model has not been tested quantitatively in risky choice.

More recently, a number of studies have relied on eye-fixations during choice between alternatives, to gain insight into the preference formation process. For example, Krajbich, Rangel and colleagues have shown that an extension of the Drift Diffusion Model (*DDM*; [12,13]), the attentional *DDM* (*aDDM*), accounts well for observed preferences between consumer products, food items and 50–50 monetary gambles [21–24]. To do so, the *aDDM* assumes that the value of the sampled alternative is modulated by eye-fixations, so that the values of the non-fixated alternatives are attenuated compared with the fixated ones. In the domain of risky choice, a number of studies have contrasted within-alternative and within-attribute models, and reported partial support for both [20,24–28]. In particular, Glöckner and Herbold [20] analyzed risky choice while monitoring eye-movements, and provided evidence against the *PH* model and in favor of the *PCS* and *DFT* models (see also [29] for similar results). Finally, in a recent investigation of eye-movements during risky choice, Stewart, Hermens, & Matthews [30] concluded that, while eye-movements contribute to choice preference, this contribution is mostly independent of the values sampled. In other words, the more one looks at an alternative the more likely s/he is to choose it, independently of the magnitude of amount or probability.

**Fig 1:**
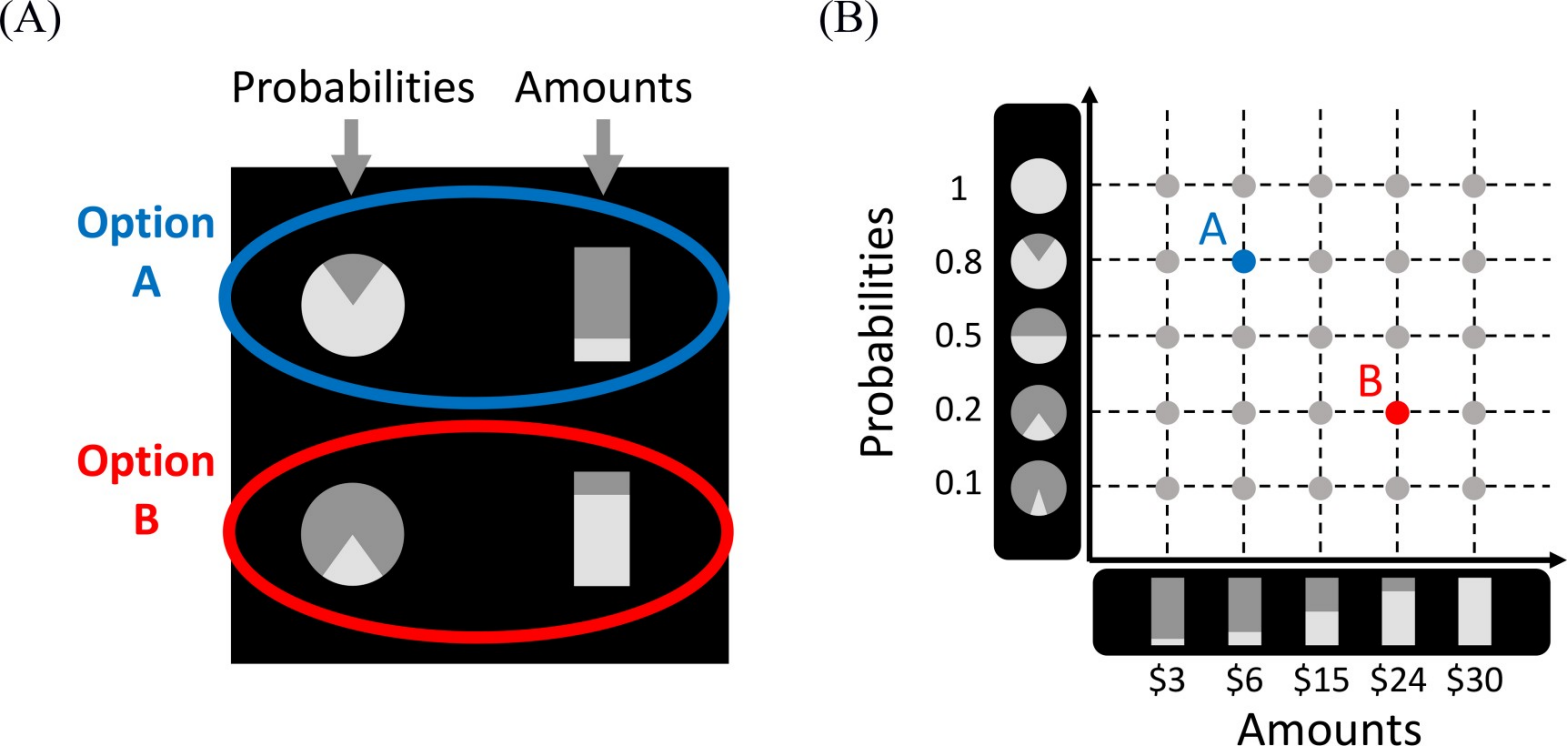
Stimuli and study design. (A) Example of the stimuli used in the experiment. Amounts were represented by the lower (brighter) parts of divided bar graphs, and probabilities by the lower (brighter) sectors of pie charts. Note that the figure is not to scale, and the colored ellipses and labels are shown for illustration purposes only (they were not used in the actual experiment). (B) Choices were drawn from a 5x5 two-dimensional grid with amounts along one dimension, and probabilities along the other. The two stimuli from panel A are shown in this grid. Choice stimuli were presented without deadline until response.

The aim of the current study is to develop and contrast process models of risky choice, which are constrained by the eye movements of participants making decisions. In particular, we adopt an integration-to-boundary framework, which allows to predict both choices and their decision-time, and we extend the *aDDM* [21,22,31] approach to the domain of risky choice (see also [24] for a recent extension to 50–50 monetary gambles). In this regard, a central question is whether the preferences are formed by integrating global alternative-values, based on multiplicative interactions between amounts and probabilities (*within-alternative* processing), or by sampling and integrating attribute-comparisons (*within–attribute* processing*)*. Furthermore, using process models that include attentional modulation of fixated information, we wish to account for individual differences in risk preference. While previous work has highlighted the impact of task-complexity (e.g., number of alternatives and attributes) in determining the decision strategy adopted by the participants (e.g., [32]), here we focus on the simplest type of risky choice (between pairs of alternatives, each consisting of a probability *p* to win amount *x*, see Fig 1A). Thus, our aim here is not to determine which of these two types of processes prevail in any choice scenario (both can take place, subject to task-conditions and individual differences). Rather, we wish to test if, at least for this simple case, the more normative (within-alternative and multiplicative) strategies are within the capacity of participants resources. Towards this end, we carry out a systematic investigation of risky choice with simple two-outcome lotteries, while eye-fixations are monitored. To anticipate our results, we provide a clear demonstration that within-alternative and multiplicative evaluations are being used, subject to individual differences that correlate with choice normativity.

## Results

The participants were tested on choices between simple lotteries of the type (*x*_*1*_ with *p*_*1*_ and otherwise 0, vs. *x*_*2*_, with *p*_*2*_ and otherwise 0; where *x*_*1*,*2*_ are monetary amounts and *p*_*1*,*2*,_ are the corresponding probabilities of winning). The choice problems (94 trials) were selected by systematically sampling a two-dimensional grid of probabilities and amounts (Fig 1B). Dominated choice problems (in which both the amount and probability of one option were higher than in the other option) were excluded except for 10 catch-trials, which were used to assess task engagement. To discourage numerical calculations, the choice alternatives were presented in graphical format (Fig 1A). The experiment was incentive compatible: it was explained to the participants that one of their choices will be randomly chosen and played out for real money at the end of the experiment (see S1 Methods and S3 Fig for details on the stimuli and task instructions).

### Choice behavior

**Fig 2:**
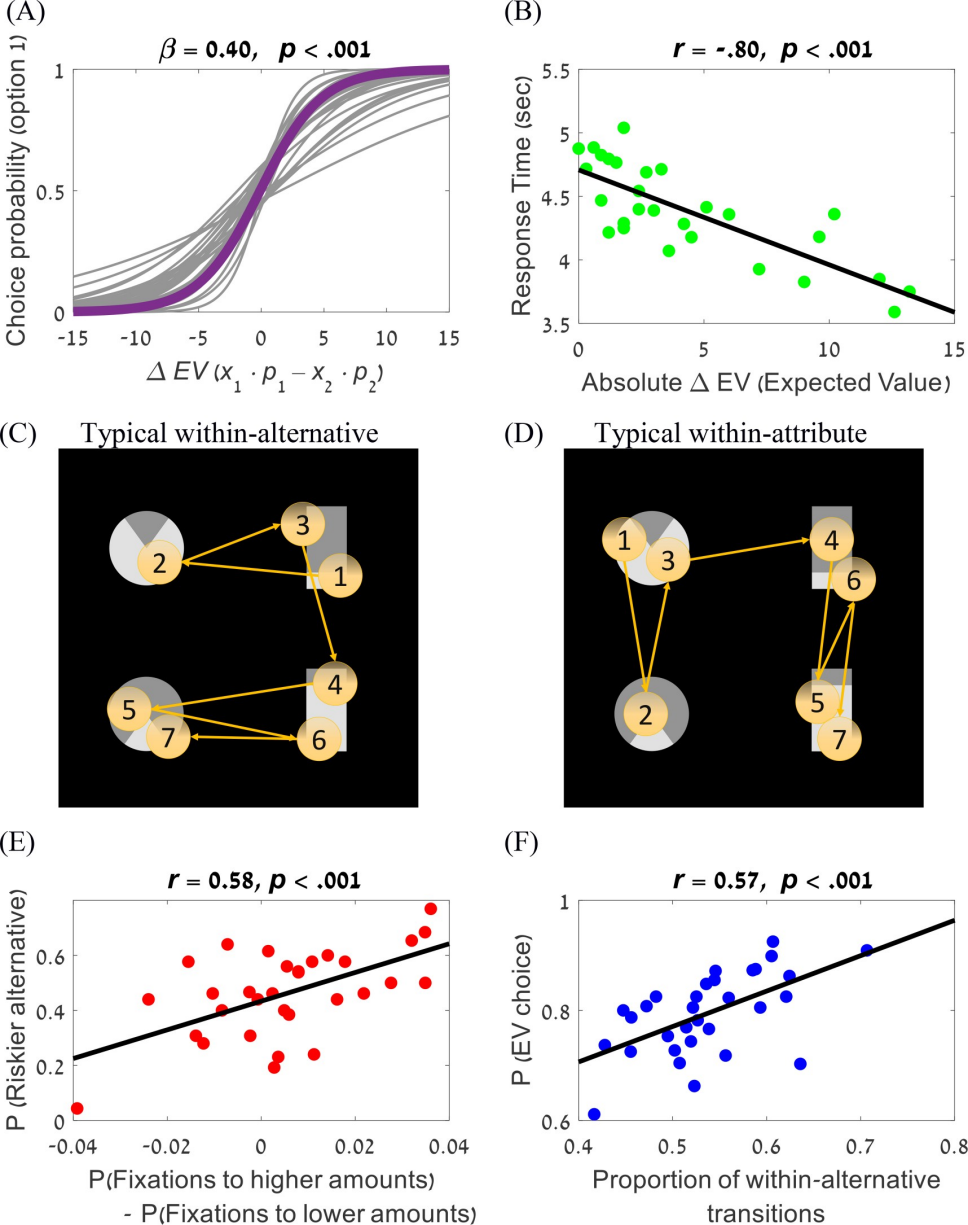
Choice and eye-movements analysis. (A) The participants were sensitive to EV differences between the options. Solid purple line corresponds to the group fit; grey lines correspond to the fit of individual participants. (B) Response times were negatively correlated with the alternatives' EV differences. (C-D) Example eye-trajectory characterized by within-alternative transitions (in C) and by within-attribute transitions (in D). The numbers indicate the order of fixations. (E) The difference in proportion of fixations to higher and lower amount was correlated with risk-seeking preference. (F) The proportion of within-alternative transitions correlated with the proportion of higher EV choices.

We began by examining the basic psychometric properties of our choice-data. Analysis of the "catch-trials" showed that the participants chose the better option (higher in both amount and probability) in 97% of these trials (*SD* = 6%). Next, we conducted a mixed-effect logistic regression on the choice data, with the Expected-Value (*EV)* differences (*x*_*1*_*∙p*_*1*_ *–x*_*2*_*∙p*_*2*_) as a predictor, and with random intercepts and slopes at the participant level. The results indicated that the participants were sensitive to *EV* differences, and preferred lotteries with higher *EV*s over lotteries with lower ones (*β* = 0.40, *p* < .001; Fig 2A). Additionally, using a Pearson correlation analysis, we showed that the reaction time (RT) of a decision decreased as the absolute *EV* difference between the lotteries increased (*r* = -0.8, *p* < .001; Fig 2B). This finding is consistent with previous process models such as the *PCS* [20], the *aDDM* [21], and the *DFT* [16], indicating that the participants take longer to decide when the evidence (as measured by the *EV*-difference) is smaller.

Finally, we evaluated the risk-preferences of the participants. To this end, we focused on choice problems with similar *EV*s (*|*Δ*EV|* ≤ 1, *N*_*choice problems*_ = 26), and examined the proportion of trials in which high-payoff/low-probability lotteries (riskier options) were preferred over low-payoff/high-probability lotteries (safer options). Following the *CPT* regularity of differential risk-attitudes for low vs. medium/high probabilities (see S1 Text), we examined the risk-preferences separately for these two probability domains: i) low-probability cases, in which one of the lotteries has p < .25 (e.g., $24 with p = .1 vs. $6 with p = .5), and ii) high-probability cases, in which both lotteries have p ≥ .25 (e.g., $30 with p = .5 vs. $15 with p = 1); the .25 cutoff was selected to match *CPT* (see S1 Text). A paired samples *t*-test indicated that, consistent with *CPT*, the participants showed higher levels of risk-aversion for medium/high probabilities as compared to low ones (*t*(30*)* = 3.84, *p* < .001). Follow-up one-sample *t*-tests (against .5) indicated that the participants showed risk-aversion for medium/high probabilities (*t*(30*)* = 4.49, *p* < .001); no risk-aversion, however, was obtained for low probabilities (*t*(30*)* = -0.11, *p* = .9).

### Eye-fixations and individual differences

On average, the participants made 9.05 fixations (*SD* = 3.56) per trial, with a mean duration of 407ms (*SD* = 244 ms) per fixation. Also, on average across participants, there was no significant difference between the proportion of fixations towards amounts and probabilities (*t*(30) = 0.78, *p* = .44). There was, however, a remarkable difference between participants in this proportion, which was correlated with participants’ risk preferences: the more a participant fixated on amounts, the more likely he or she was to choose the riskier alternatives (*r* = .48, *p* = .006; S1A Fig). To understand this relationship we examined individual differences in fixating the higher of two amounts/probabilities, as this can explain risk-biases (looking more at higher amounts or at lower probabilities leads to risk-seeking according to the *aDDM* [21,22,24]). Importantly, we find that the more a participant tends to fixate on amounts the more s/he fixates on the larger of them (*r* = .47; *p* = .007; S1B Fig), and similarly for probabilities (*r* = .46; *p* = .007; S1C Fig). Finally, the frequency of fixations on the higher of the two amounts was positively correlated with risk-seeking (*r* = .58; *p* < .001; Fig 2E), and the frequency of fixations on the higher of the two probabilities was negatively correlated with risk-seeking (*r* = .45; *p* = .01;S1D Fig) see also [24,33].

We also examined the eye-trajectories in relation to their transitions between the four attributes (*x*_*1*_, *p*_*1*_, *x*_*2*_, *p*_*2*_). The transitions between decision attributes (amounts and probabilities) were classified into three categories [20,25,30]: i) Within-alternative transitions–transitions between attributes that belong to the same alternative. ii) Within-attribute transitions–transitions between different alternatives, within the same attribute. iii) “Diagonal” transitions–transitions between the amount of alternative A and the probability of alternative B and vice versa. Fig 2C and 2D show one example each for within-alternative and within-attribute trials, respectively. An Analysis of Variances (ANOVA) revealed significant differences of the transition probabilities between the three transitions types (*F*(2,60) = 431.1, *p* < .001). Post-hoc comparisons showed that the participants made more within-alternative than within-attribute transitions (*p* < .001), as well as more within-attribute than diagonal ones (*p* < .001). The proportion of within-alternative transitions (out of all transitions) was subject to individual differences and was correlated with *EV*-choice performance (Δ*EV)*, such that the higher the fraction of within-alternative transitions the higher was the proportion of the alternative with the higher *EV* to be chosen (*r* = .57, *p* < .001; Fig 2F).

### Predicting choices using eye-fixations

**Fig 3:**
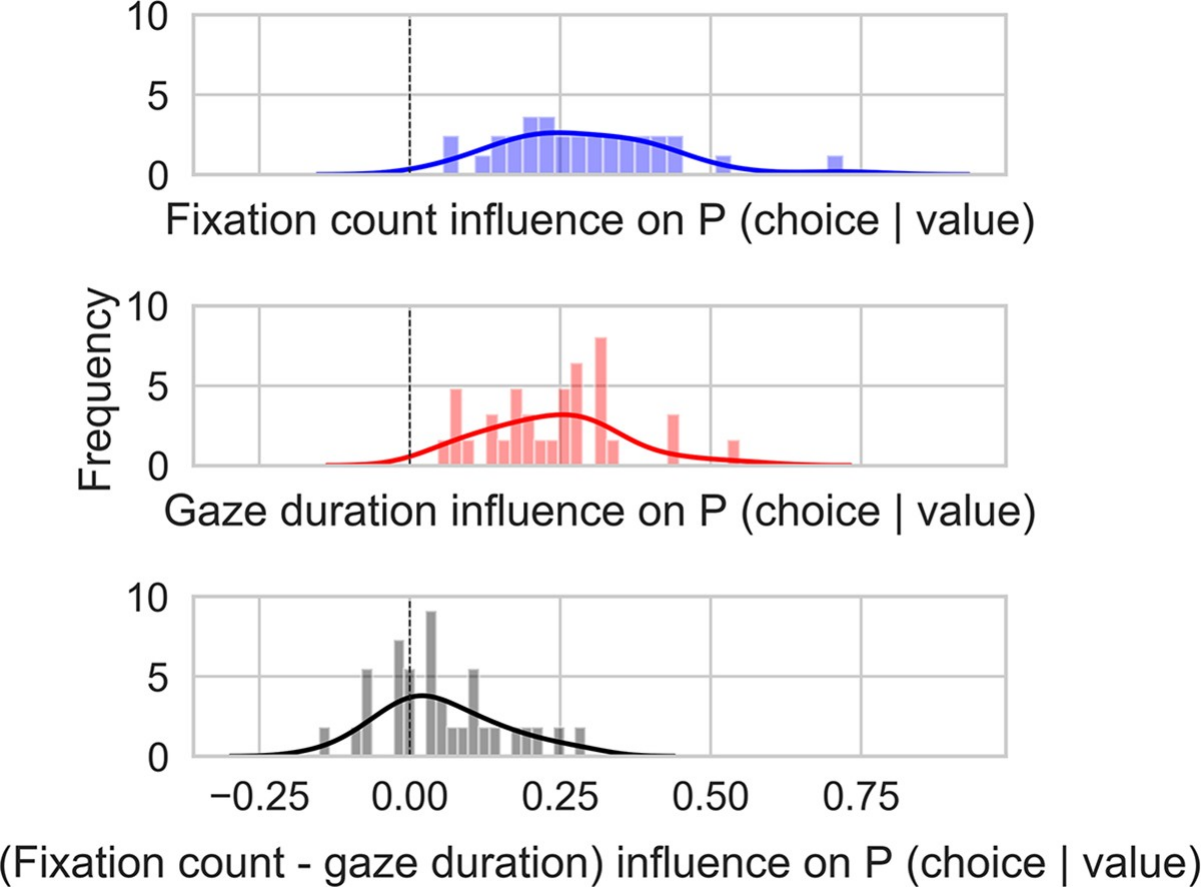
Gaze-influence on choice (after accounting for values), for the fixation count (upper panel; blue), for the total-gaze duration (middle panel; red) and their difference (lower panel; grey).

Recent research has demonstrated that attentional mechanisms play a key role in the development of preferences [24,34–38]. In particular, it was shown that the more an alternative is fixated on, the more likely it is to be chosen [21,30,39]. We first estimated the benefit of looking time (or of the number of fixations) on choice, using a measure that was developed in another study ([40]). Specifically, using logistic regression we predicted the choices from the *EU*-difference between the two alternatives (with parameters fitted for each subject on all his/her choices). Then, for each trial, we computed the deviation between the actual choices (coded as 0 or 1), and the probabilities predicted by the *EU*-difference. This residual was averaged separately for trials in which the choice had a positive or a negative gaze-advantage (we did this twice, for total gaze duration and for number of fixations). We then computed the difference between these measures to obtain the average difference in choice probability for the items with a positive versus negative final gaze advantage, when corrected for the influence of their (*EU*) values. As shown in Fig 3 (see also [40] for similar results on non-risky choices), there is a marked gaze-advantage in predicting the choice. At the group level, this advantage has a mean value of 0.29 (*SD* = 0.14) for the number of fixation (Fig 3, upper panel), and a mean value of 0.24 (*SD* = 0.11) for the total gaze duration (Fig 3, middle panel). Finally, we found a small, but significant prediction enhancement, for the number of fixation predictor (*t*(30) = 2.87, *p* = 0.008; Fig 3, lower panel).

Second, we have confirmed the impact of gaze on choice, using a multiplicative computation of the alternatives' values based on *EU* (or *CPT*) values and a monotonic function of gaze-time (or number of fixations; see S2 Text for details).

**Fig 4:**
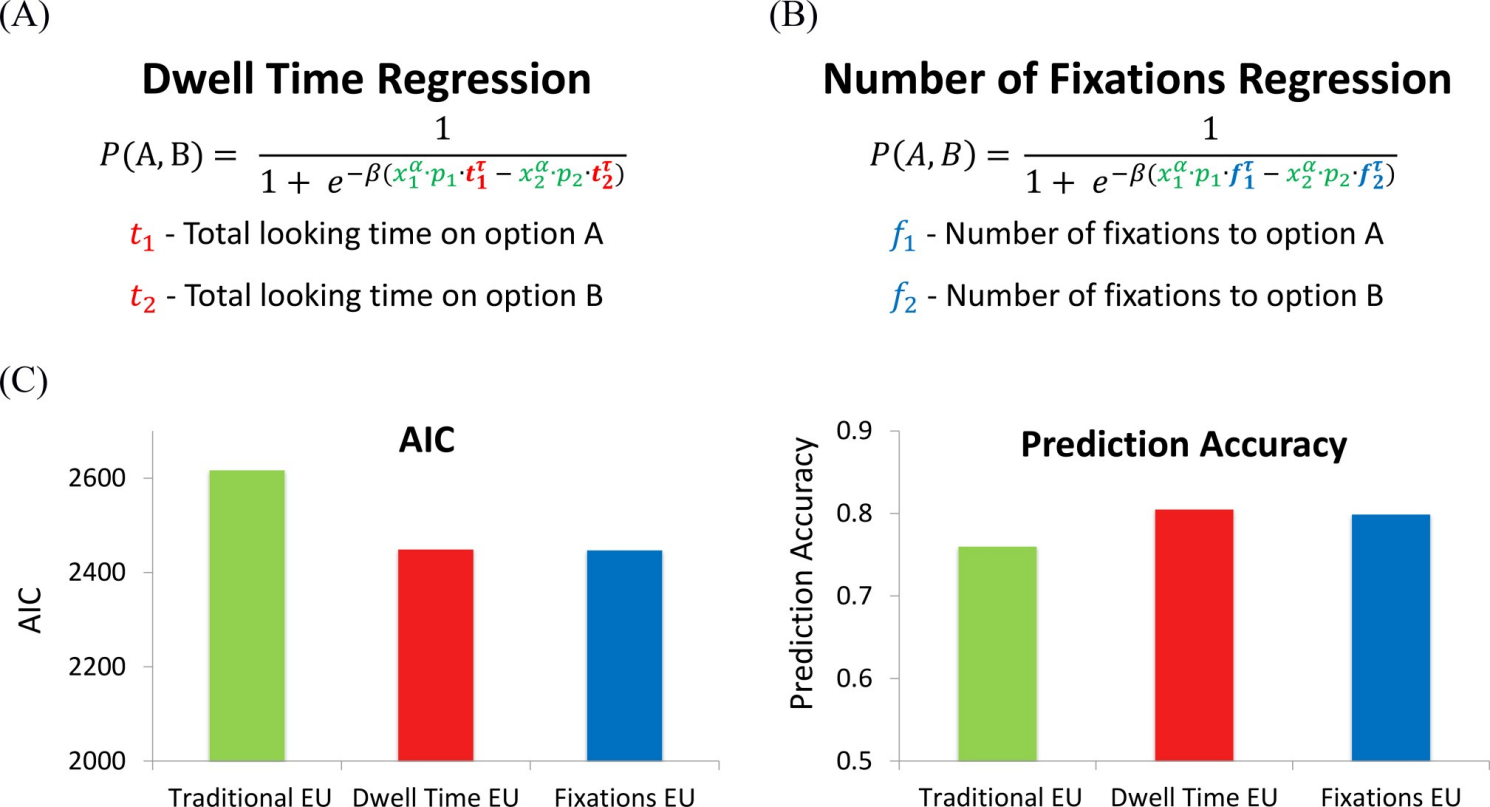
Expected Utility based regression models. (A) EU_Dwell-time_: the EU value of each of the alternatives is modulated by the total looking time on each alternative. (B) EU_Fixations_: the EU value of each of the alternatives is modulated by the relative number of fixations towards it. (C) Prediction accuracy and AIC for the traditional, dwell time and number of fixations EU.

**Fig 5:**
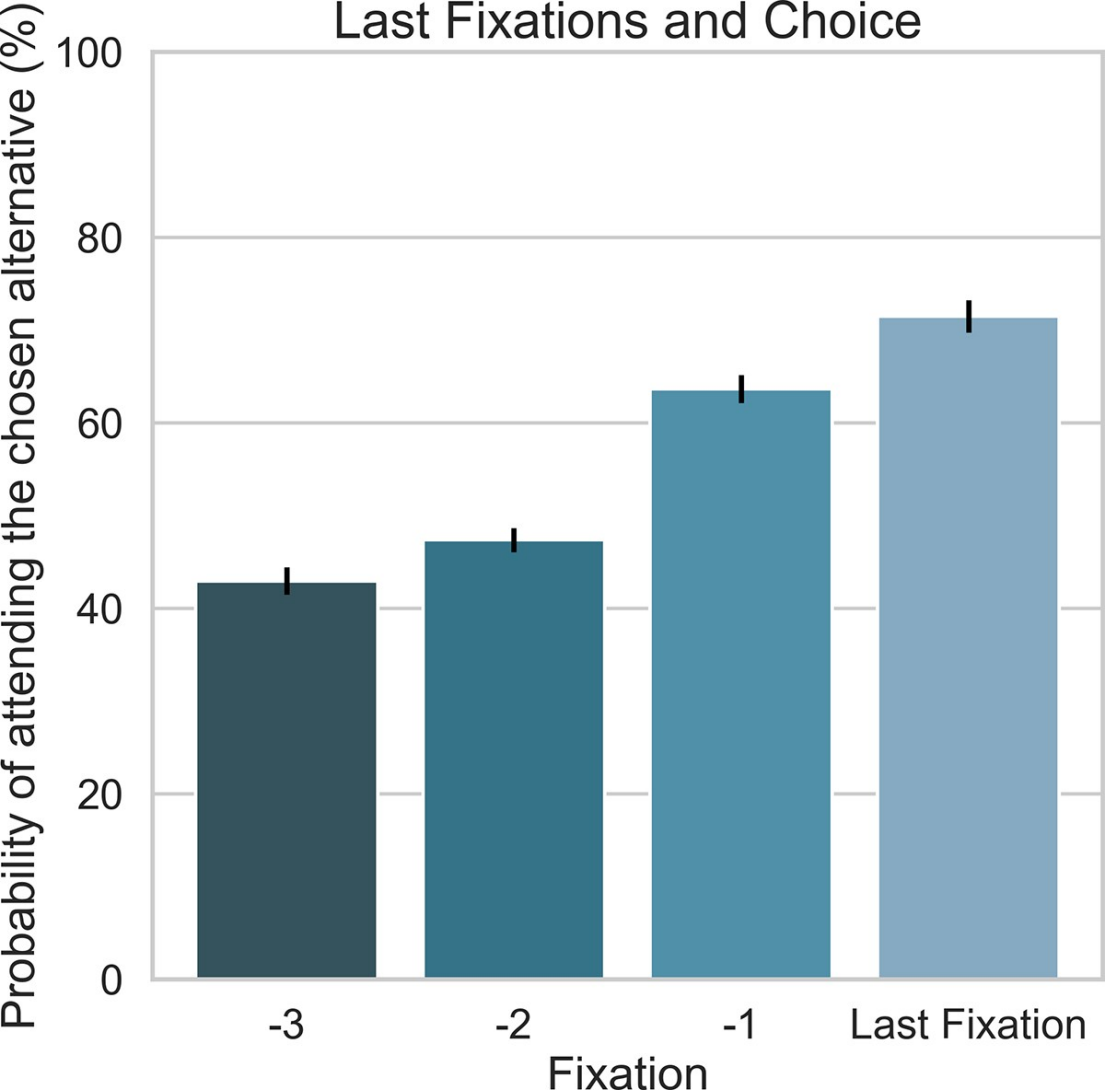
The probability of fixating on an attribute of the chosen alternative as a function of the last four fixations. Error bars correspond to standard errors of the mean.

As illustrated in Fig 4A, we examined an *EU × time* regression model (similar conclusions were obtained for *CPT* based models, see S2 Text), in which the *EU* value of each alternative increases with its dwell time on the two alternatives (*α* is the risk-parameter of *EU*, *τ* is a saturation parameter, and *β* is a slope parameter). Additionally, we examined a similar regression model, in which dwell-times were replaced with the number of fixations each alternative is sampled (Fig 4B). Comparison of these models with the traditional *EU* (which does not take eye-movements into account) showed that using eye-movements significantly improved prediction accuracy and *AIC* comparted with the traditional *EU* (Fig 4C). Note also, that the prediction accuracy and *AIC* which were obtained using the number of fixations, equal (for *EU*) or surpass (for *CPT*), the prediction accuracy and *AIC* obtained using the measure of dwell-time. In addition, in both gaze based regressions (number of fixations and dwell time), the fitted values of saturation-parameter *τ* were lower than 1, indicating that, for example, looking twice as long at an alternative increases its value by a factor of less than 2. One way to understand this non-linear saturation is in relation to a leak of the accumulated values [14,41,42]. In such leaky integration models, the accumulated evidence saturates at an asymptotic value, and remains constant even if more integration time is allowed. Accordingly, at each fixation one samples and accumulates a value, however, as the trial proceeds the accumulated value leaks, resulting in a type of recency. Indeed, the percentage of match between the fixated alternative and the final choice as a function of fixation number (backwards from the end) showed a clear recency pattern (Fig 5; note that an *aDDM* model without leak can also generate a recency pattern [43], therefore a quantitative model comparison is needed to determine if leak is required to account for the actual pattern).

### Towards a process model of risky choice based on eye-movements

The central aim of this study is to develop and contrast two classes of process models that differ in the way attentional (or eye) transitions affect the integration of amounts and probabilities. Both types of models assume that: a) fixated objects receive enhanced attention, b) attention modulates the weight of value integration [21], and c) recently sampled values are weighted more than earlier ones [14,41,42]. The models differ, however, on how the values are integrated into preferences. Note that we do not aim to test specific models but rather distinguish between broad classes of models based on certain principles, in particular, between *within-attribute* vs. *within-alternative* models [20,25,32,44]. While the former is used in models such as *PH* and *DBS*, the latter is used in models such as *EU*, *CPT* and *PCS*. We also examined a more hybrid model, which still relies on multiplicative within-alternative computations, but also allows some extent of competition between the attributes.

#### Within-attribute integration models

Models from this class assume that when decision-makers attend to one attribute (e.g., amount or probability), they accumulate the value-difference (or categorical difference) of the two alternatives on that attribute, according to:

$$Y_{A}(t+1)={(1-\lambda )∙Y}_{A}(t)+D_{A}(t)$$

$$Y_{B}(t+1)={(1-\lambda )∙Y}_{B}(t)+D_{B}(t)$$

where *Y*_*i*,_ i∈{A,B} is the accumulated preference for alternative i, *λ* is an integration-leak factor that emphasizes recent values, and *D*_*i*_ is the value (or categorical) difference between the attributes, which depends on eye-fixation and model variant (see S3 Text *Within-attribute integration models* for a detailed description of the models). This mechanism was implemented in two model variants. In the first one, preferences were generated by accumulating the normalized differences (min-max normalization, over the whole set of decision problems) of the attended attribute values. For example, if the participant had to choose between A:($20, 0.2) and B:($10, 0.5), then the difference between the normalized amount values (of $20 and $10, respectively) is accumulated whenever the representations of amounts are fixated (see S3 Text *within-attribute integration models*). Likewise, the difference between the normalized probability values (of 0.2 and 0.5, respectively) is accumulated whenever the representations of probabilities are fixated. The second model assumes integration of categorical differences; this follows the *DBS* assumption that people have access to ordinal comparisons rather than values [18]. Therefore, in the above example, the accumulator associated with alternative A increases by one unit at each fixation of an amount (since $20 is more than $10), and the accumulator of alternative B increases by one unit at each fixation of a probability (since 0.5 is larger than 0.2). This means that the mechanism accumulates binary counts of comparison between the same attribute in different alternatives [17,19]. To enhance these models’ performance we allowed an additional parameter: attentional modulation, which enhances the weight of sampled attributes ([21,22]; see S3 Text *within-attribute integration models)*. Note that since the values of both attributes are used in the comparison, these models assume either the existence of some degree of peripheral vision, or reliance on memory. Since memory cannot play a role during the first fixation of an attribute (and since peripheral vision is less sensitive to the low contrast of our stimuli in any fixation, including the first), we assumed that default values (mid-range of the amounts and probabilities values used in the experiment) are used for the yet un-scanned attributes. The default values were replaced with the actual attributes' values at the first fixation to each attribute. This treatment was implemented in all versions the process models (see S3 Table).

#### Within-alternative integration models

The second class of models assumes that the values that are integrated are associated with the alternatives and are multiplicatively formed from the attributes (as in expected utility). This mechanism was also implemented in two models. The first model has single-layer architecture and involves two accumulator units, one for each alternative (A or B). On each fixation, the accumulators are updated with the integrated subjective utilities of the fixated alternative (which is based on multiplication of the subjective-amounts and subjective-probabilities; see S3 Text *Within-alternative selection/One-layer leaky accumulators*), according to:

$$Y_{A}(t+1)={(1-\lambda )∙Y}_{A}(t)+{SU}_{A}(t)$$

$$Y_{B}(t+1)={(1-\lambda )∙Y}_{B}(t)+{SU}_{B}(t)$$

where *Y*_*i*,_ i∈{A,B} is the accumulated preference of alternative-i, *λ* is an integration-leak, and *SU*_*i*_ is the subjective expected utility of alternative-i (similar to *CPT*), subject to attentional modulation that depends on eye-fixation. As in the *aDMM* model [21], this model assumes that the inputs are modulated by gaze direction (i.e., higher weight is assigned to the fixated alternative than to the non-fixated one). Note that in this model the update does not depend on whether the current fixation is on amount or probability, but only which alternative is fixated, with the non-fixated alternative being attenuated. For example, when one looks at either the amount or the probability of alternative A, the corresponding accumulator is updated with the integrated subjective utility of that alternative, while the other accumulator is updated with an attenuated value (which can be zero) of the subjective utility of alternative B.

**Fig 6:**
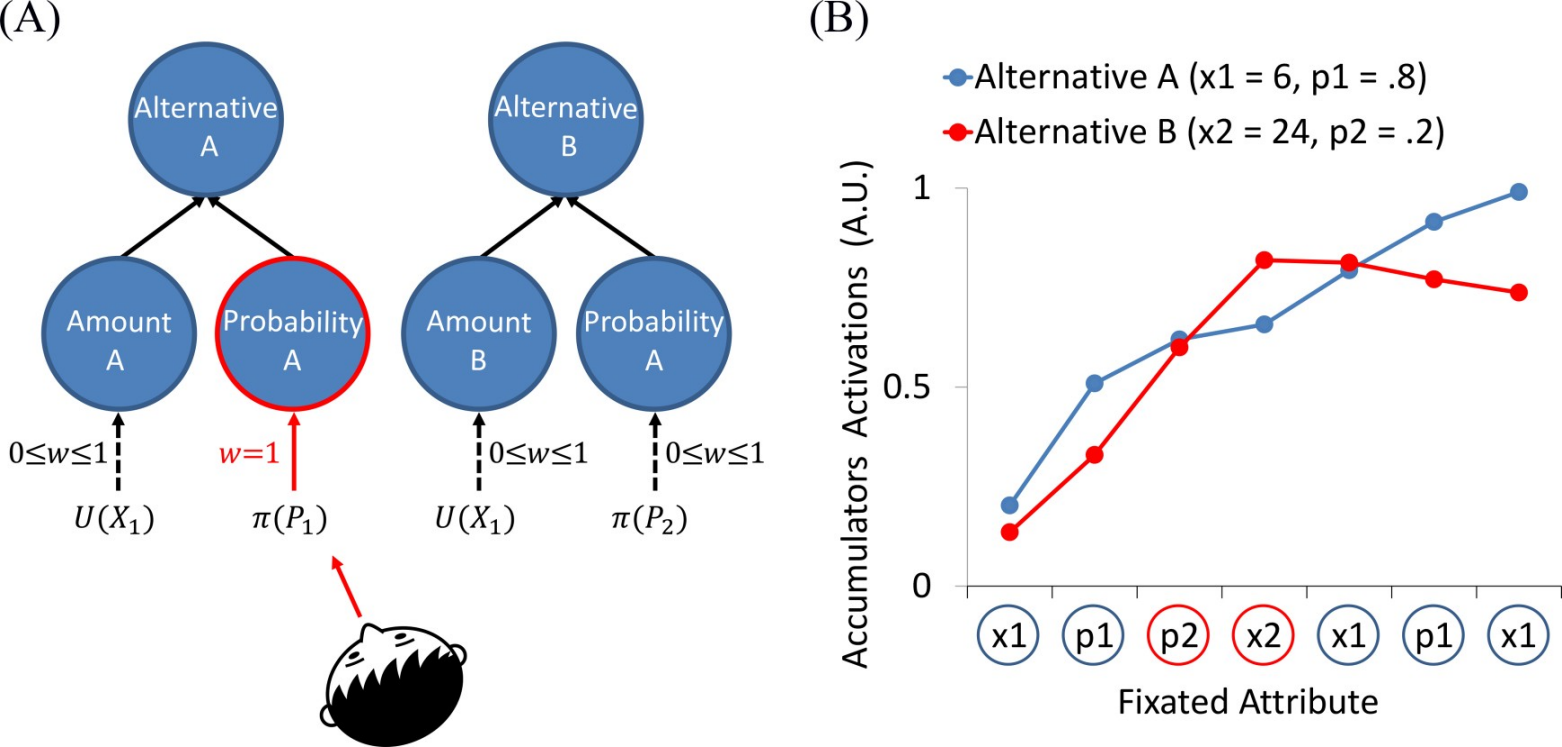
Illustration of the two-layer leaky accumulator model and its dynamics. (A) The first layer consists of four leaky-accumulators associated with the different attributes, and the second consists of two leaky accumulators associated with the alternatives’ values. The units in the first layer are updated with the attentionally modulated subjective values of each attribute (the red arrow indicates the input from the attended attribute, whereas the black arrows indicate the attenuated inputs from the unattended attributes). The units in the second layer are fed with the first layer units' activations, and accumulate their product. (B) Simulated run of the two-layer leaky accumulators model using the average best fitted parameters (see S2 Table), in a choice between: A($6, .8) and B($24, .2); A-wins. Blue circles (x-axis) correspond to fixation toward A, and red circles correspond to fixation toward B. Values on the y-axis correspond to the activations of the second-layer accumulators.

The second within-alternative model contains two-layers of leaky-accumulators in cascade (Fig 6A); as we will show, this model allows to apply attentional modulations to specific attributes and not only to the whole alternative. The first layer of the model consists of four leaky-accumulators associated with the four different attributes (*x*_*1*_, *p*_*1*_*; x*_*2*,_ *p*_*2*_). Unlike in the previous (single layer) version, these units are updated with the attentionally modulated subjective values of each *attribute*. For example, when a participant looks at the amount of alternative A (i.e., *x*_*1*_), the accumulator of that attribute is updated with the subjective value associated with it (*x*_*1*_^*α*^, where *α* is a free parameter), while the other accumulators (of *p*_*1*_, *x*_*2*_ and *p*_*2*_) are updated with attenuated subjective values of their attributes. The second layer of the model consists of two leaky-accumulators corresponding to the integrated preference of the two alternatives. At each fixation, each second layer (alternative) accumulator is fed with the activations of the first layer units associated with it, by accumulating the product of their values (see Fig 6B for illustration of the model dynamics and S3 Text *Within-alternative selection/Two-layer leaky accumulators* for details). In one version of the two-layer model, we also introduced mutual inhibition between the amount units (competition between *x*_*1*_ and *x*_*2*_) and the probability units (competition between *p*_*1*_ and *p*_*2*_). One can think of such a model as implementing a hybrid between within-attribute and within-alternative processes: while the alternatives units still receive multiplicative input from both their attributes units, the mutual inhibition (depending on its strength) can polarize the difference in activation, subject to attentional modulation based on the current fixation.

The models were fitted to the data in two steps. In the first, we fitted the models to choice data, based on the values of the alternatives and the eye-fixations made for that decision, using maximum likelihood estimations to obtain the best model parameters (see S1 Methods). In the second step, we used the models with their fitted parameters from step 1, to make predictions for decision-time, under an integration-to-boundary framework (in which we included a new set of parameters that correspond to the response boundary). At this stage we also compared the models on their ability to predict both choices and decision-times. We wish to note that the level of activation-leakage (in all models) is a free-parameter, so that the case in which *λ* = 0, corresponds to the more standard Drift-Diffusion models, and is thus explicitly tested as part of our model fit procedures.

In addition to the process models, we also fitted a number of benchmark non-integration to boundary models. Specifically we examined traditional compensatory models, such as *EV*, *EU* and *CPT*, as well as non-compensatory heuristic models such as *Maximax* [45], *Least-Likely* [46] and *PH* ([9]; see S3 Text *Heuristics* for detailed description of these models).

To evaluate the models' capabilities to fit the data, we used several selection criteria: prediction-accuracy, *AIC* and cross-validation measures (see S1 Methods *Model Selection*). For the heuristic models, whose choices are deterministic, we only examined their accuracy measures [8]. Because it can be argued that the prediction-accuracy of models with fixed (or no) parameter values (such as the heuristic models) cannot be compared to the prediction-accuracy of models with fitted parameter values [47], we compare them using the Cross-Validation/Accuracy measure.

### Step-1: Choice data

The most complex of the models (in terms of number of parameters) is the within-alternative process model, which has four free parameters. The first two, *α* and *γ* correspond to the *CPT* parameters for risk aversion and probability weighting [3], respectively, *θ* corresponds to the *aDDM* attentional modulation parameter, and *λ* is the activation-leak. As we show in S5 Fig, we carried out a recovery exercise, showing that our fitting procedure is able to provide a good recovery for all those parameters over a wide range of values that correspond to those found in the actual data. This non-trivial result is helped by the fact that our 94 choice problems systematically span the choice space.

As shown in Table 1, the *within-alternative* process models with attention modulation and leak gave the best fit and showed the highest cross-validation prediction accuracy. They outperformed both the within-attribute process models, as well as the traditional, non-integration to boundary models (compensatory and non-compensatory heuristics). These results speak against the hypothesis that the participants accumulate only the differences of the attended attributes. We also found that the within-attribute models with perfect (rather than leaky) integration (Normalized and Binary differences), resulted in much worse *AIC*, prediction accuracy, and cross-validation (therefore in Table 1 we report only the within-attribute models which include leak as a free parameter). We note that the within-alternative choice models required a significant degree of information leak (*λ*_group_ = 0.58). As shown in S3 Table, we explicitly tested four versions of within-alternative models that include an attentional modulation but no activation-leak, all of which resulted in much poorer prediction-accuracy and *AIC* fit values. By contrast, the leaky within-alternative process models outperformed (on prediction accuracy, *AIC* and cross-validation) the regression models that include either *EU* or *CPT* together with the number of fixations (see S2 Text and Table 2). This suggests that considering dynamic processes, such as attentional shifts and leak of activation improves prediction accuracy and fit measures beyond what is achievable by using only the number of fixations. Note that the within-alternative two-layer leaky accumulators model outperforms the single-layer accumulator model. This result suggests that the perception of the attributes is dynamic and is subject to modulation by attentional processes. Finally, the hybrid model resulted in fits (*AIC* and prediction accuracy) that did not exceed those of the within-alternative model (see S3 Table), and with a moderate mutual inhibition value (.13) which does not trigger a full all-or-none dynamics. Due to its complexity, we leave a full investigation of this model to future research.

**Table 1:** Model comparison.

*Model*	*AIC*	*Prediction* *Accuracy*	*Cross-Validation* *(-2∙LogLikelihood)*	*Cross-Validation (Accuracy)*
***Traditional Models***				
*EV*	2789	75.1%	567	75.1%
*EU*	2617	76.5%	527	76.0%
*CPT*	2364	81.2%	484	79.6%
***Heuristics***				
*MaxiMax*	-	44.8%	-	44.8%
*Least-Likely*	-	55.2%	-	55.2%
*Priority Heuristic*	-	58.3%	-	58.3%
***Fixation based regression model****s*				
*EU*_*Fixations*_	2447	79.9%	506	78.7%
*CPT*_*Fixations*_	2173	83.9%	453	81.7%
***Within-attribute Integration***				
*Normalized differences*	2716	76.8%	551	75.4%
*Categorical differences*	2724	76.4%	576	74.4%
***Within-alternative Integration***				
*one-layer leaky accumulators*	1980	86.1%	445	83.8%
**two-layer leaky accumulators**	**1877**	**87.2%**	**436**	**84.1%**

AIC values are rounded to the nearest integers. Bold entry indicates the best fitting models. Note that AIC differences exceeding 10 are considered very strong evidence in favor of the model with the lower numerical values.

**Table 2:** Models of risky-choice.

***Traditional Models***
*Expected Value (EV)* • Participants choose the alternative with the higher Expected-Value: *EV* = *x*∙*p*.
*Expected Utility (EU)* • Participants choose the alternative with the higher Expected-Utility: *EU* = *u*(*x*)∙*p*; *u*(*x*) = *x*^*α*^.
*Cumulative Prospect Theory (CPT)* • Participants choose the alternative with the higher Subjective-Utility: $SU=u(x)∙\pi (p);\;\pi (p)=\frac{p^{\gamma }}{{\left(p^{\gamma }+{\left(1-p\right)}^{\gamma }\right)}^{\frac{1}{\gamma }}}.$
***Fixation based regression model****s*
*EU*_*Fixations*_ • *U*_*alternative*_ = *u*(*x*)∙*p*∙*n*^*τ*^, where *n* is the number of fixations to the alternative, and *τ* is a saturation parameter.
*CPT*_*Fixations*_ • Defined analogously to *Multiplicative EU*_*Fixations*_, but with *p* replaced with *π*(*p*) according to *CPT*.
***Heuristics***
*MaxiMax* • Choose the alternative with the highest maximum amount.
*Least-Likely* • Choose the alternative with the lowest probability of its worst outcome.
*Priority Heuristic* • Compare sequentially the following attributes: minimum amounts, probability of minimum amounts, maximum amount. • Stop when difference between the attributes reaches a termination criterion.
***Within-attribute Integration***
*Normalized differences* • Normalize the amounts and probabilities using min-max normalization: $y\prime =\frac{y-min(y)}{max(y)-min(y)}$ • On each fixation, the normalized differences of the attended attribute are accumulated. Unattended attribute are underweighted. • Example: if one fixates on *x*_1_, accumulator A increases by: *x*_1_′−*θ*∙*x*_2_′, and accumulator B by: *θ*∙*x*_2_′−*x*_1_′. *θ*∈ [0,1] represents attentional modulation.
*Categorical differences* • This model was implemented as the *normalized differences* model, except that instead of accumulating normalized differences, the model accumulates counts based on categorical comparisons. • Example: if one fixate on *x*_1_ and *x*_1_>*θ*∙*x*_2_, accumulator A increases by one unit, and B remains the same. *θ*∈ [0,1] represents attentional modulation.
***Within-alternative Integration***
*One-layer leaky accumulators* • On each fixation, this model accumulates the *SU* of the two alternatives, defined as in *CPT*. • At fixation toward alternative A, the input of alternative B is attenuated (and vice versa). • Example: if one fixates on *x*_1_, accumulator A increases by: *SU*_*A*_, whereas the accumulator B increases by: *θ*∙*SU*_*B*_. • The activations of the alternatives' accumulators are subject to leak.
*Two-layer leaky accumulators* • Two layers of leaky accumulators, the first estimates subjective amounts and probabilities (defined as in *CPT*), and the second estimates integrated *SU*. • On each fixation, the first-layer units are updated with the subjective amounts and probabilities, with the inputs of the unattended attributes attenuated. •Example, if one fixates on *x*_*1*_ the inputs of *p*_*1*_, *x*_*2*_ and *p*_*2*_ are *θ*∙*π*(*p*_1_), *θ*∙*u*(*x*_2_) and *θ*∙*π*(*p*_2_), respectively. The second-layer units are fed with the activations of the first layer units, and accumulate the product of their values. • The units of both layers (first and second) are subject to leak.

**Fig 7:**
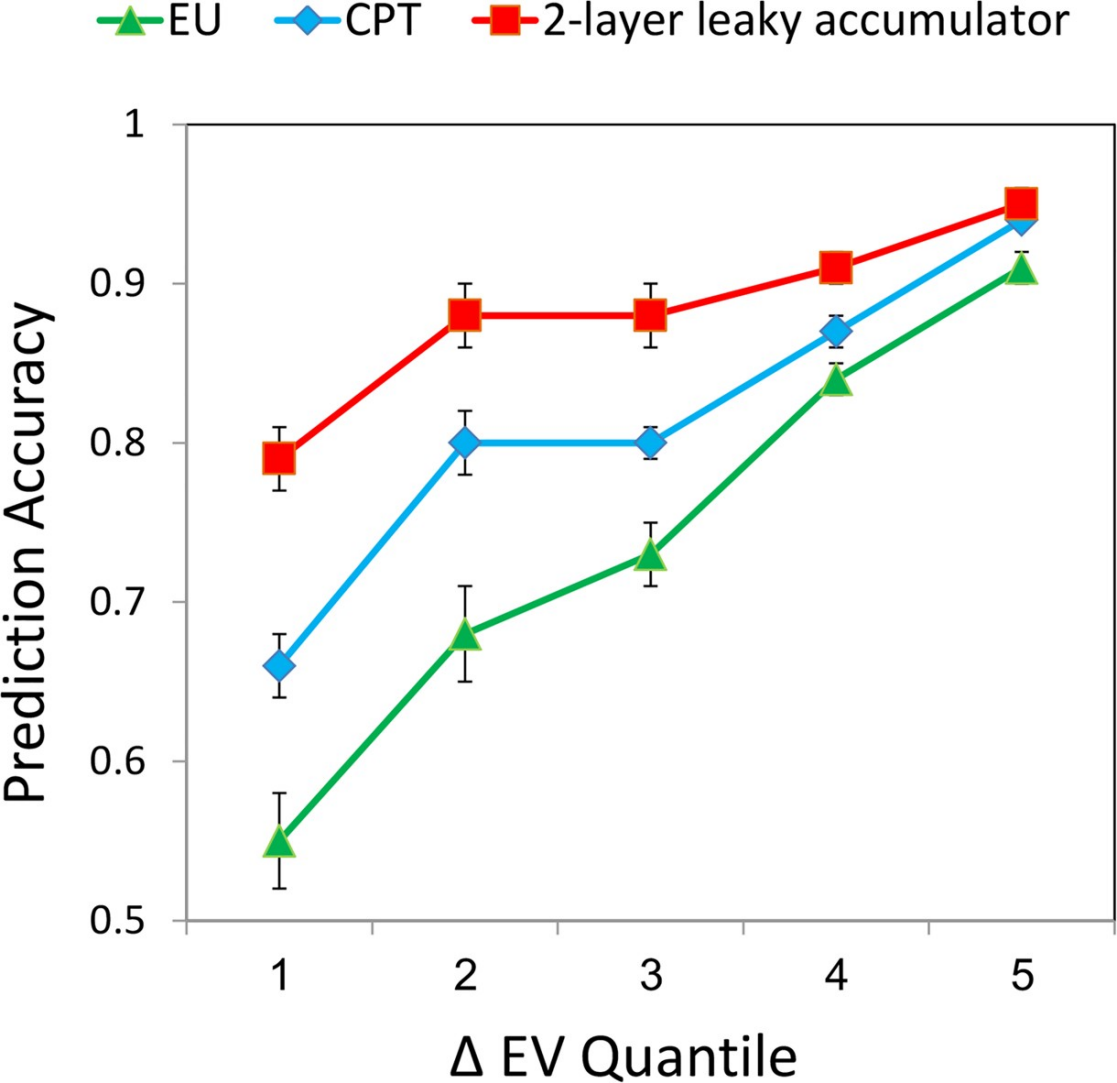
Predictive accuracy of the EU, CPT and the two-layer within-alternative integration models as a function of ΔEV quantile. The two-layer model outperformed all other models especially in difficult decisions (low EV-differences). Bars denote S.E., clustered by subjects.

Finally, we carried out a comparison of the predictive accuracy of our best performance model–the two-layer leaky accumulators—with that of the traditional *EU* and *CPT* models across all decisions as a function of *EV*-differences. The comparison demonstrates that the difference in prediction accuracy is especially large for difficult choices (low *EV*-differences, 1–3 Quantiles; Fig 7), suggesting that attentional modulations are particularly significant in difficult decisions [48].

### Step-2: Accounting for both choice and decision-time

We contrasted the *within-alternative* and the *within-attribute* models, in accounting simultaneously for choices and decision-times. To this end, we adopt an integration-to-boundary framework, which assumes that preferences are accumulated until they cross a decision criterion [49,50]; this introduced a few more parameters (for the boundary) into the model (see S1 Methods *Model Fitting*). The models are now set to estimate the probability of a subject’s choice conditioned on its decision time and fixations. This probability is accumulated for all choice trials of the participant to a total likelihood, which is used to optimize the boundary parameters. Two families of decision boundaries were tested, for each of the models: i) the standard fixed (time-invariant) boundary, which introduces a single new boundary parameter, and ii) a collapsing (time-variant) boundary model, which introduces three new parameters (see S1 Methods *Optimization procedure*: *choices and decision-times*, for further details regarding the implementations of these two types of models). The collapsing boundary model has been the focus of recent investigations in decision neuroscience [41, 42], and appears to be favored in experimental tasks that span over longer time intervals (more than 2–3 sec [51–53], but see [54] for an alternative explanation based on across-trial variability parameters).

**Fig 8:**
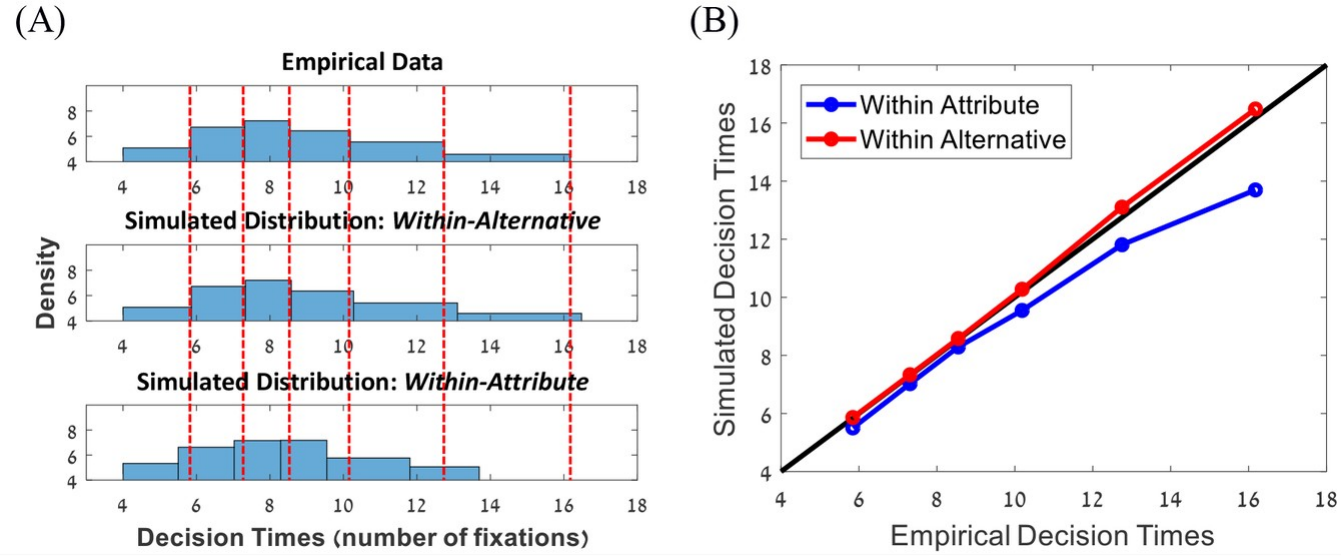
Accounting for choices and decision times. (A) Group quantile (Vincentizing; [12]) decision-time density distribution (in number of fixations to decision). Dashed red lines indicate the quantiles of the actual data distribution ([.1, .3, .5, .7, .9, .99]). (B) Group Quantile-Quantile plot comparing the actual decision time and the simulated decision time of the within-alternative (red) and within-attribute models (blue). Note that the within-alternative model captures better the tail of the distribution.

The results show that, with both decision boundary families, the two-layer leaky accumulator model outperformed all the other models. Among the two types of boundary families, the best fits were obtained for the collapsing boundary models (*AIC* and cross-validation), despite the cost of the two extra parameters. For this reason, we only report below the results for this type of boundary (see S4 Table for comparison of all within-attribute and within-alternative models using fixed and collapsing boundaries). We find that the within-alternative/two-layer leaky accumulator model (*AIC* = 14,492) decisively outperformed the within-attribute/normalized differences model (*AIC* = 15,815; Δ*AIC* = 823), in accounting for decision-times (conditioned on the actual fixation patterns). Finally, we used these models to predict the distribution of decision times (measured in number of fixations), for novel but statistically matched patterns of fixations. To this end, for each trial we simulated a fixation sequence that is based on a statistical model of the participant’s fixations towards the four attributes as a function of their values [21,30]. The results indicate that for the two-layer leaky accumulator model, the predicted and actual decision-time distributions show a good match, however for the normalized differences model, the tail of the predicted decision-time distribution deviates from that of the actual decision-time distribution (Fig 8).

### Accounting for individual differences in risk-bias and in normative choice

**Fig 9:**
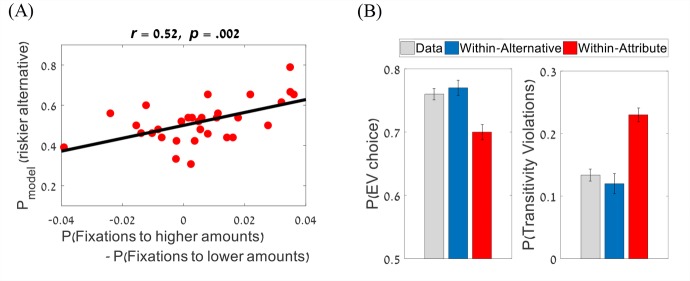
Model predictions and individual differences. (A) The proportion of fixations toward the higher amount was correlated with the risk-seeking preference predicted by the two-layer within-alternative integration model with parameters fixed at the group-mean, but with actual fixations. (B) The within-alternative model is closer to the data and predicts a higher fraction of EV-choices (left panel) and a lower fraction of (irrational) transitivity violations (right panel), compared with the within-attribute model. Error bars represent the standard error of the mean.

Our best within-alternative integration model accounts also for the empirical correlation we reported between the proportion of fixations a participant makes to the higher of the two amounts and his or her risk-preference bias (Fig 2E; see also [24]). To show this, we simulated choices for each participant, based on his or her fitted model-parameters and the participant's actual fixation sequence. The correlation between the model's risk-preference prediction and the proportion of fixations to the higher amount (*r* = .58, *p* < .001), was exactly equal to the empirical correlation obtained in the data (Fig 2E). Next, we sought to demonstrate that this relation is associated with the fixation pattern and not merely with differences in model parameters. To this end, we simulated choices for each participant, by using his or her actual fixation sequences, however, this time we used model parameters that correspond to the group mean (rather than the individually fitted parameters). This resulted in a significant correlation (*r* = .52; *p* = .002; Fig 9A) between the risk-preference and the proportion of fixating on the higher amount. This correlation between risk-biases and fixation-pattern relies upon the model’s attentional component, which gives higher weights to the attributes on which the participant fixates. For example, assume that a participant is asked to choose between A:($20, 0.5) and B:($10, 1). If s/he fixates more the amount of alternative A than the amount of alternative B, higher weights would be given to the former, and thus the riskier alternative (A) would be preferred by the model over the safer one (B).

Finally, we address an important question: which preference-formation mechanism (*within-alternative* or *within-attribute*) results in better normative performance, and thus can be regarded as more adaptive? To answer this, we simulated the two types of models based on the participants' best fitted parameters and actual fixation sequences, and we examined two measures of normative choice predicted by each model: i) the fraction of *EV-*choices (for simplification we discuss normativity in terms of *EV*, but the same would hold in terms of *EU*), and ii) the fraction of transitivity violations–a direct measure of choice irrationality ([55,56]; see S4 Text). As seen in Fig 9B, the normative performance is higher for the within-alternative model than for the within-attribute model, for both measures: *EV*-choices: *t*(30) = 6.27, *p* < .001 and transitivity violations: *t*(30) = 5.15, *p* < .001. This is expected because our within-alternative model, like *CPT*, assumes a multiplication between subjectively transformed amounts and probabilities, which also maintains choice-consistency. Although the normative model requires a multiplication of objective values whereas our model requires a multiplication of subjective values, this discrepancy is relatively minor compared with non-multiplicative strategies (i.e., within-attribute integration or heuristics). Moreover, we have found that the more within-alternative transitions a person makes, the higher is his or her fraction of *EV* choices (Fig 2F; see also [28]). This correlation can be naturally understood, since the participants rely on a within-alternative multiplicative mechanism, and this operation is likely to be more precise following an actual transition between amounts and probabilities (i.e., a fixation on one attribute of an alternative followed immediately by a fixation to the other attribute of the same alternative), than following a non-direct transition (where one of the to-be-multiplied attributes is based on memory or defaults). Consistent with this, we found a correlation across participants between the prediction accuracy of the *within-alternative model* and the proportion of within-alternative transitions (*r* = 0.39, *p* = .03).

## Discussion

The main aim of our study was to elucidate the mechanisms by which different attributes (amounts and probabilities) are integrated to generate an overall subjective value of choice alternative. To this end, we focused on choices between simple lotteries and developed process models of risky choice, which are constrained by eye-fixations and we assumed a fixation-based attentional modulation. In addition, we introduced activation-leak and examined two types of decision-boundaries, in order to account for decision times. Within these models we specifically contrasted *within-alternative* multiplicative models and *within-attribute* type models, and carried out a systematic parametric investigation of choices between simple lotteries (*x*_*1*_ with *p*_*1*_ vs. *x*_*2*_ with *p*_*2*_), while tracking participants' eye-fixations.

First, we replicated previous findings indicating that participants prefer lotteries with higher *EV*s. In particular, the choice probability of the alternative with the higher *EV* increases (and choice-RT decreases) with the *EV*-difference between the lotteries. Nevertheless, participants also exhibited risk biases that are probability-dependent, being risk-averse at high/medium probabilities, but not at low probabilities. Second, we found that, on average, the eye-scan patterns were dominated by within-alternative as compared to within-attribute or diagonal transitions (Fig 2C–2D, respectively), and that individual differences on this eye-scan pattern correlate with *EV*-choice (see also [28], for a similar result). Third, we used eye-fixations to constrain a number of process models that accumulate preference across fixations, using an *aDDM* approach with two attributes [21,57]. Here we contrasted two types of integration-to-boundary process models: i) within-attribute models, and ii) within-alternative models. As shown in Table 1, the latter resulted in the best predictive accuracy and measures of fit. Importantly, the two-layer model also accounted well for decision times (Fig 8) and for individual differences in risk biases (Fig 9). Finally, the worst performance in our task was obtained for the non-compensatory heuristic models. For example, the best of the heuristics (the *PH*) resulted in a worse fit than even the simple *EV* model (see cross-validation measure in Table 1).

The conclusions favoring the within-alternative multiplicative models may need to be qualified for the task conditions we used here. First, we used simple lotteries with single non-zero outcomes (*x* with *p*, 0 with *1-p*). It is possible that the amount of within-attribute (non-compensatory) processing would increase when more complex choices are used [25,32]. While we cannot rule out this possibility, recent research in the domain of probabilistic inferences [58–60] and risky choice [20,61], indicates that when decision processes are monitored via eye-tracking (which does not slow down the decision process) rather than via mouse pressing techniques (e.g., [10]), participants are able to use compensatory strategies for relatively high complexity levels (see also [62] for a multi-attribute choice task). Second, our results need to be qualified to the use of analog (graphic rather than symbolic) representation of the data. We used this format of representation to reduce the possibility that our participants (who are students that may be familiar with *EV*-principles, and are required to do 104 choice problems), adopt an explicit *EV* calculation strategy. We believe that such a strategy is less likely with analog information, and thus our results favoring an implicit multiplicative mechanism are even more remarkable. Third, the alternatives in our experiment were aligned only vertically (one lottery was placed over the other, with the amounts and probabilities placed left/right, see Fig 1A). It is possible to argue that this layout favors within-alternative processing (left to right or right to left transitions). Note, however, that a horizontal layout (left/right alternatives) triggers a strong bias favoring within-attribute processing, in particular, comparing the horizontally aligned amount bars. Nevertheless, we report in S4 Fig data from a pilot Experiment (*N* = 13) using a horizontal alternative layout, which shows that even under such within-attribute favorable conditions, we still find dominance for within-alternative transitions. Here we only wish to support the following conclusion: humans possess the capacity to spontaneously deploy an ‘economic’ (multiplicative across-dimension) type computation (which is analog rather than symbolic; [63]), supporting the idea that humans are closer to normative principles than previously thought (see also [60,64]). Future research will be needed to further quantify to what extent the use of this mechanism (or strategy) is contingent upon task complexity and stimuli type.

There are several important properties of our winning process model that we want to highlight. First, it assumes two layers of leaky accumulators, one for the estimation of subjective amounts and of subjective probabilities, and the one for the evaluation of the integrated subjective values (the combination of subjective amounts and probabilities). Second, it assumes that the units in the second layer are updated via a multiplication of the activation of the corresponding, first layer units (Fig 6A). This is similar to how the *CPT* model generates subjective utilities. In fact, we find a high correlation between the utility function’s curvature parameter (*α*) of the classical *CPT* and the corresponding parameter of our process model (*r* = .91, *p* < .001), with higher *α* -values for the classical *CPT* (see S2 Fig). This suggests that the classical *CPT*-parameters reflect a combination of several processes, such as attention allocation and subjective-value transformation [65]. Note also, that the model assumes an activation-leak, a feature that allows it to account for recency effects in the data (see Fig 5), and prevents a double-integration that would occur in the two-layer model in its absence. Third, in addition to predicting choices, the model also predicts decision times, describing the preference formation dynamics under the integration to boundary framework with inputs that correspond to a multiplicative transformation of subjective amounts and probabilities. In particular, we found support for a collapsing boundary, consistent with a recent normative analysis of value based decisions [66], and with choice studies that span longer intervals [51–53].

Other process models of risky choice, such as *DFT* [15,16] also assume an implicit multiplicative interaction between amounts and probabilities. In *DFT*, however, this is not due to the multiplication of amounts and probabilities but rather to the sampling frequency of the amounts, which changes with the corresponding probabilities. This implies that observers look (or attend) more to a given amount if the corresponding probability is higher. In our data, while we find that the relative number of fixations to an amount increases with its probability, this increase was quite minor (about 1%), and therefore cannot explain the multiplicative interaction [20]. However, it is possible that eye-fixations under-estimate the differential of covert attention modulation.

Future research is also needed to better understand the neural mechanisms underlying these computations [67–69]. While the computation of subjective amounts and probabilities can be understood to involve simple psychophysical transformations over amounts (unbounded scale; [70]) and probabilities (bounded scales; [71]), the nature of the multiplicative interaction between neural activations requires future investigations. Note that a multiplicative interaction is also assumed in the *PCS* risk model [20]. To do so, *PCS* had to assume different neural substrates for amounts (neural activations) and for probabilities (synaptic weights). The latter assumption, however, may be difficult to justify for one-shot decisions, which allow little opportunity for learning synaptic weights. We thus suggest that the multiplicative interactions involve neural activations. While less standard than linear interactions [72], a number of neural mechanisms have been proposed to mediate multiplication of neural activity in neural systems [73,74]. Future research is also needed to extend the scope of this investigation from simple lotteries to more complex ones (with multiple outcomes) and from binary to multiple choices.

## Methods

### Ethics statement

The experiment was approved by the ethics committee at Tel-Aviv University (1321253), consent was given by a written form.

### Experiment

#### Participants

35 Tel-Aviv University undergraduate students (24 females; ages range from 19 to 26, Median age = 23) were recruited to the experiment. All of them reported having normal or corrected-to-normal vision. Four of the participants were not able to carry out the eye tracker calibration task, and thus did not take part in the main experiment, leaving 31 participants. The participants received course credit in exchange for participating, as well as a bonus fee ranging from 0 to 30 Israeli Shekels (ILS), which was contingent upon their choices.

#### Apparatus

Eye-movements were recorded using a Tobii TX300 desk-mounted eye-tracker (23" monitor with 1920 x 1080 pixels resolution, sampling rate: 300Hz, spatial accuracy: 0.5°), attached to an Intel i7 personal computer. Displays were presented using Psychtoolbox for MATLAB [75]. Viewing distance was approximately 60 cm. Responses were collected via the computer keyboard. A chin rest was not used.

#### Stimuli

Each choice consisted of two simple lotteries in the form of *p*_*1*_ chance to get *x*_*1*_ ILS (otherwise nothing) vs. *p*_*2*_ chance to get *x*_*2*_ ILS (otherwise nothing). An example of the display is presented in Fig 1A. Amounts were represented by the lower parts of divided bar graphs, and probabilities were represented by the lower sectors of pie charts. These attributes appeared at the vertices of an imaginary square subtending 14.5° (15.25 cm), and located in the center of a black screen. The height of each bar graph subtended 2.07° (2.17 cm) and its width subtended 0.67° (0.69 cm); the radius of each pie chart subtended 0.67° (0.69 cm). Thus, the bar graphs and pie charts had exactly the same surface. The alternatives were aligned vertically (i.e., one lottery was placed over the other, with the amounts and probabilities placed left/right, Fig 1A); but see the Discussion and the results of a pilot study reported in S4 Fig, in which we used horizontal alignment. On each trial, the left/right positions of the amounts and probabilities were randomly determined.

#### Choices

Choice problems were constructed in the following way: we generated a 2-dimensional grid with amounts (3, 6, 15, 24 and 30 ILS) along one dimension, and probabilities (0.1, 0.2, 0.5, 0.8 and 1) along the other dimension. The resulting grid contained 25 lotteries (Fig 1B), each of which was paired with all other possible lotteries. Stochastically dominated choices (in which both the amount and probability of one alternative were higher than those of the other) were excluded, except for 10 choice problems which served as "catch-trials". Overall, the experiment consisted of 104 separate choices: 94 non-dominated trials and 10 "catch-trials" (all the choice problems are given in S1 Table).

#### Procedure

The participants signed an informed consent form prior to the experiment. Then, a calibration of the eye-tracker took place. In case the calibration was successful, the experiment started, otherwise recalibration was performed. At the beginning of the experiment, instructions were given to the participants (see S3 Fig). The experimenter emphasized that choices should be made in accordance with subjective preferences and that there is no "correct" choice. The experiment consisted of two blocks of 52 choice trials each. A short break was allowed between blocks, and a recalibration procedure was performed before the second block. Each trial began with a fixation display which consisted of a red 0.2° × 0.2° fixation cross (+) that remained on screen until a continuous fixation of 500 ms duration was made. Then, the two lotteries were presented until response. Choice was made using the up and down arrow keys. Participants were told that after completing the experiment, one of the choices will be randomly chosen and payed out. The whole experiment took approximately 30 min per participant. Choice order as well as the horizontal position (left/right) of the amounts and probabilities were randomized for each participant; the vertical position of each lottery (up/down) was randomized between subjects.

#### Eye-movements

Fixations were classified as being directed to a certain attribute, if they were within 100 pixels of the center of that attribute and lasted at least 50 ms. Two consecutive fixations to the same attribute were joined and considered as one fixation. Trials longer than 10 sec or shorter than 500 ms (4% of all trials), as well as trials in which the participants did not look at all of the attributes (4% of all trials), were excluded from further analysis.

### Models of risky-choice

Here we briefly describe the key features of the models applied (for a full description see S3 Text*)*. In all of the models (except for the Heuristics models), the probability of choosing each alternative is calculated using an exponential version of Luce’s choice rule [76,77]:

$$P(x_{1},p_{1};x_{2},p_{2})=\frac{1}{1+e^{-\beta (U_{1}-U_{2})}}$$

where *U*_1_ and *U*_2_ are the utilities of the alternatives, and *β* is a free parameter indicating the sensitivity of the model to their difference.

## Supporting information

S1 Table Choice problem used in the experiment. (PDF)

S2 Table Best fitting parameters of the risky choice models. (PDF)

S3 Table Additional model variations. (PDF)

S4 Table Process model comparison. (PDF)

S1 Text Cumulative Prospect Theory (CPT) risk attitudes predictions. (PDF)

S2 Text Predicting choices using eye-fixations. (PDF)

S3 Text Models of risky choice. (PDF)

S4 Text Transitivity Violations. (PDF)

S1 Methods Supplementary experimental methods. (PDF)

S1 Fig Eye-fixations and individual differences. Correlations between the proportion of fixations toward amounts/probabilities and risk-attitudes. (A) The proportion of fixations toward amounts was positively correlated with preference for the riskier alternative. (B) The proportion of fixations toward amounts was positively correlated with the tendency to fixate on the larger amount. (C) The proportion of fixations toward probabilities was positively correlated with the tendency to fixate on the larger probability. (D) The difference in proportion of fixations to higher and lower probabilities was negatively correlated with risk-seeking preference.
(TIF)

S2 Fig Relationship between the models' utility parameters. The utility curvature parameters (α) of our process model and of the CPT model were highly correlated across participants. However, the CPT parameters have higher values (the solid black line corresponds to the identity line). This may suggest that the CPT parameters reflect a combination of different components, such as selective allocation of attention and subjective value transformation [65].
(TIF)

S3 Fig Experimental instructions. At the beginning of the experiment the above instructions were presented to the participants. The experimenter read them out loud and verified that the participants fully understand the task. The text translates from Hebrew as follows: "Hello! In the following experiment you will be asked to choose between different lotteries. Each lottery holds a different amount of prize money and different chances of winning. You will be asked to choose which of two lotteries you prefer. At the end of the experiment, one of the lotteries would be randomly chosen, and you will get the chance to play and win real money. Yellow arrow towards the upper bar: the amount of money when the bar is full is 30 Israeli shekels. Yellow arrow towards the lower pie chart: the chance of winning the prize when the entire pie is full is 100%. Use the ↑ and ↓ arrows to choose which lottery you prefer".
(TIF)

S4 Fig Pilot study. In a pilot study conducted before the main experiment, the lotteries were displayed side by side (i.e., horizontal alignment) with the bars presented always on top and the pie-charts presented always underneath. In this study, the participants made more within-alternative than within-attribute transitions (as in the experiment reported in the main text).
(TIF)

S5 Fig Parameters recovery. The ability of the fitting procedure to accurately identify the parameters of our best-fitting model (within-alternative/two-layer leaky accumulators) was tested by simulating the model using the participants’ estimated parameters. For each parameter, we simulated the model for the 94 non-dominated experimental trials. The figure shows the generative parameters of the model plotted against the recovered parameters, for (A) α-risk aversion (B) γ-probability weighting (C) θ-attentional modulation and (D) λ-activation leak for simulated data.
(TIF)

S1 References (PDF)
