# Supplementary material for: The formation of preference in risky choice
Source: PLoS Comput Biol. 2019 Aug 29;15(8):e1007201. doi: 10.1371/journal.pcbi.1007201 (PMC6738658; doi:10.1371/journal.pcbi.1007201)
Supplement: S1 Table — (PDF) [file pcbi.1007201.s001.pdf]

S1 Table. Choice problem used in the experiment.

Problem	x_1	p_1	x_2	p_2	$\lvert\Delta EV\rvert$	*Domination*
1	3	0.2	6	0.1	0	-
2	3	0.5	15	0.1	0	-
3	3	1	30	0.1	0	-
4	6	0.5	3	1	0	-
5	6	0.5	15	0.2	0	-
6	6	0.8	24	0.2	0	-
7	6	1	30	0.2	0	-
8	15	0.2	3	1	0	-
9	15	0.2	30	0.1	0	-
10	24	0.1	3	0.8	0	-
11	24	0.5	15	0.8	0	-
12	24	1	30	0.8	0	-
13	30	0.1	6	0.5	0	-
14	30	0.5	15	1	0	-
15	6	0.2	3	0.5	0.3	-
16	15	0.1	6	0.2	0.3	-
17	6	0.1	3	0.1	0.3	✓
18	3	1	24	0.1	0.6	-
19	6	0.5	3	0.8	0.6	-
20	6	0.5	24	0.1	0.6	-
21	15	0.2	3	0.8	0.6	-
22	24	0.1	15	0.2	0.6	-
23	24	0.1	30	0.1	0.6	✓
24	30	0.1	3	0.8	0.6	-
25	6	0.1	6	0.2	0.6	✓
26	6	0.1	3	0.5	0.9	-
27	15	0.1	3	0.2	0.9	-
28	3	0.8	15	0.1	0.9	-
29	24	0.1	3	0.5	0.9	-
30	24	0.2	6	1	1.2	-
31	30	0.2	6	0.8	1.2	-
32	6	0.2	3	0.8	1.2	-
33	24	0.1	6	0.2	1.2	-
34	3	1	15	0.1	1.5	-
35	6	1	15	0.5	1.5	-
36	15	0.2	3	0.5	1.5	-
37	15	0.1	6	0.5	1.5	-
38	30	0.1	3	0.5	1.5	-
39	30	0.2	15	0.5	1.5	-
40	6	0.2	3	1	1.8	-
41	6	0.2	6	0.5	1.8	✓
42	30	0.1	6	0.2	1.8	-
43	3	0.2	24	0.1	1.8	-
44	6	0.1	3	0.8	1.8	-
45	3	1	6	0.8	1.8	-
46	3	1	24	0.2	1.8	-

47	6	0.8	15	0.2	1.8	-
48	24	0.2	6	0.5	1.8	-
49	24	0.2	15	0.2	1.8	✓
50	24	0.2	30	0.1	1.8	-
51	30	0.1	6	0.8	1.8	-
52	3	1	6	0.1	2.4	-
53	3	0.2	30	0.1	2.4	-
54	6	0.5	3	0.2	2.4	✓
55	3	0.8	6	0.8	2.4	✓
56	24	0.2	3	0.8	2.4	-
57	24	0.1	6	0.8	2.4	-
58	24	0.1	24	0.2	2.4	✓
59	15	0.5	6	0.8	2.7	-
60	15	0.5	24	0.2	2.7	-
61	6	1	3	1	3	✓
62	6	1	15	0.2	3	-
63	6	0.5	30	0.2	3	-
64	6	1	30	0.1	3	-
65	15	1	24	0.5	3	-
66	15	0.8	30	0.5	3	-
67	24	0.5	30	0.5	3	✓
68	30	0.2	3	1	3	-
69	3	0.5	24	0.2	3.3	-
70	6	0.8	15	0.1	3.3	-
71	3	0.8	30	0.2	3.6	-
72	6	1	24	0.1	3.6	-
73	24	0.8	15	1	4.2	-
74	30	0.5	24	0.8	4.2	-
75	6	1	15	0.1	4.5	-
76	15	0.5	3	1	4.5	-
77	15	0.5	30	0.1	4.5	-
78	30	0.2	3	0.5	4.5	-
79	3	0.8	15	0.5	5.1	-
80	24	0.1	15	0.5	5.1	-
81	6	1	15	0.8	6	-
82	24	0.5	6	1	6	-
83	24	0.5	30	0.2	6	-
84	30	0.2	15	0.8	6	-
85	6	0.8	24	0.5	7.2	-
86	15	0.8	24	0.2	7.2	-
87	3	1	15	0.8	9	-
88	6	1	30	0.5	9	-
89	15	0.8	30	0.1	9	-
90	15	1	30	0.8	9	-
91	24	0.5	3	1	9	-
92	24	0.5	30	0.1	9	-
93	30	0.2	15	1	9	-
94	30	0.5	24	1	9	-
95	24	0.5	3	0.8	9.6	-

96	24	0.1	15	0.8	9.6	-
97	24	0.2	15	1	10.2	-
98	30	0.5	6	0.8	10.2	-
99	3	1	30	0.5	12	-
100	30	0.1	15	1	12	-
101	3	0.8	30	0.5	12.6	-
102	24	0.1	15	1	12.6	-
103	24	0.8	6	1	13.2	-
104	24	0.8	30	0.2	13.2	-
