# Supplementary material for: The formation of preference in risky choice
Source: PLoS Comput Biol. 2019 Aug 29;15(8):e1007201. doi: 10.1371/journal.pcbi.1007201 (PMC6738658; doi:10.1371/journal.pcbi.1007201)
Supplement: S2 Table — (PDF) [file pcbi.1007201.s002.pdf]

S2 Table. Best fitting parameters of the risky choice models.

Model	α	γ	λ	τ	θ	β
Traditional Models						
EV	-	-	-	-	-	0.49 (0.35)
EU	0.79 (0.26)	-	-	-	-	1.1 (0.87)
CPT	0.72 (0.23)	0.7 (0.1)	-	-	-	2.01 (1.37)
Traditional Models						
$EU_{Dwell\ time}$	0.79 (0.25)	-	-	0.66 (0.5)	-	1.99 (1.45)
$EU_{Fixations}$	0.78 (0.26)	-	-	0.66 (0.46)	-	1.88 (1.29)
$CPT_{Dwell\ time}$	0.71 (0.24)	0.7 (0.1)	-	0.45 (0.37)	-	2.82 (1.48)
$CPT_{Fixations}$	0.71 (0.23)	0.7 (0.1)	-	0.47 (0.31)	-	3.02 (1.53)
Heuristics						
MaxiMax	-	-	-	-	-	-
Least-Likely	-	-	-	-	-	-
Priority Heuristic	-	-	-	-	-	-
Within-attribute Selection						
Normalized differences	-	-	0.21 (0.23)	-	0.04 (0.01)	1.28 (0.85)
Categorical differences	-	-	0.25 (0.14)	-	0.27 (0.13)	1.28 (0.59)
Within-alternative Integration						
1-layer leaky accumulators	0.59 (0.24)	0.57 (0.13)	0.5 (0.29)	-	0.55 (0.29)	2.42 (1.71)
2-layer leaky accumulators	0.61 (0.25)	0.59 (0.15)	0.58 (0.22)	-	0.53 0.24	2.33 (1.78)

Note. The values in parentheses correspond to the standard deviation of the best fitted parameters across participants. Empty cells indicate cases in which a parameter was not used in the model.
