# Supplementary material for: The formation of preference in risky choice
Source: PLoS Comput Biol. 2019 Aug 29;15(8):e1007201. doi: 10.1371/journal.pcbi.1007201 (PMC6738658; doi:10.1371/journal.pcbi.1007201)
Supplement: S3 Table — (PDF) [file pcbi.1007201.s003.pdf]

S3 Table. Additional model variations.

Model	*AIC*	*Prediction-Accuracy*
One-layer leaky competing accumulators (without leak)	2742	79.0%
Two-layer leaky competing accumulators (without leak)	3127	75.7%
Two-layer leaky competing accumulators (without leak at the 1st [attributes] layer)	2579	82.2%
Two-layer leaky competing accumulators (without leak at the 2nd [alternatives] layer)	2493	81.9%
Two-layer leaky competing accumulators (without default mid-range values assumption)	1911	86.7%
Hybrid model	1951	86.1%

AIC values are rounded to the nearest integers. Bold entry indicates the best fitting models. Note that AIC differences exceeding 10 are considered very strong evidence in favor of the model with the lower numerical values.
