# Supplementary material for: The formation of preference in risky choice
Source: PLoS Comput Biol. 2019 Aug 29;15(8):e1007201. doi: 10.1371/journal.pcbi.1007201 (PMC6738658; doi:10.1371/journal.pcbi.1007201)
Supplement: S4 Table — (PDF) [file pcbi.1007201.s004.pdf]

S4 Table. Process model comparison

Model	***Fixed boundary (AIC)***	***Collapsing boundary (AIC)***
Within-alternative:<br>*Two-layer leaky competing accumulators*	16,627	14,492
Within-alternative:<br>*One-layer leaky competing accumulators*	17,054	15,863
Within-attribute:<br>*Normalized differences*	17,243	15,815
Within-attribute:<br>*Categorical differences*	17,184	16,044

AIC values are rounded to the nearest integers. Bold entry indicates the best fitting models. Note that AIC differences exceeding 10 are considered very strong evidence in favor of the model with the lower numerical values.
