# Supplementary material for: The formation of preference in risky choice
Source: PLoS Comput Biol. 2019 Aug 29;15(8):e1007201. doi: 10.1371/journal.pcbi.1007201 (PMC6738658; doi:10.1371/journal.pcbi.1007201)
Supplement: S1 Text — (PDF) [file pcbi.1007201.s005.pdf]

S1 Text. Cumulative Prospect Theory (CPT) risk attitudes predictions.

The *CPT* model predicts different risk-attitudes for low and medium/high probabilities [1,2]. To illustrate this, we calculated the Certainty Equivalents (*CE*) of the *CPT* model for lotteries in the form of ($100, *p;* $0, *1-p*). This *CE* is shown in the figure below for the standard *CPT* parameters [1] of $\alpha = 0.88$ and $\gamma = 0.61$ (see also S3 Text for details about the specific *CPT* implementation which we used). One can see that the *CPT* predicts risk-seeking for lotteries with p lower than .25, whereas for p larger than .25 the model predicts risk-aversion. For that reason we used a cut-off of .25 in our risk-seeking analysis (section 2.1 in the main text).

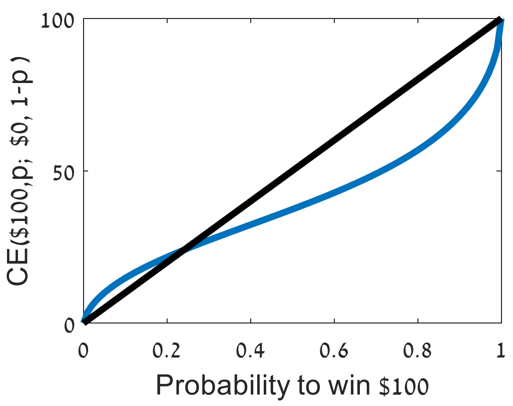

The Certainty Equivalent of the CPT model for lottery in the form of ($100, *p;* $0, *1-p*), *as a function of probability to win $100. The CPT predicts risk-seeking for low-probabilities and risk-aversion for medium/high probabilities.*
