# Supplementary material for: The formation of preference in risky choice
Source: PLoS Comput Biol. 2019 Aug 29;15(8):e1007201. doi: 10.1371/journal.pcbi.1007201 (PMC6738658; doi:10.1371/journal.pcbi.1007201)
Supplement: S2 Text — (PDF) [file pcbi.1007201.s006.pdf]

S2 Text. Predicting choices using eye-fixations

Several recent studies have shown that the more one look at an alternative, the more likely this alternative will be chosen [3–5]. Here, we examined whether this pattern holds in our data, by predicting the probability of choosing alternative $A(x_1, p_1)$ over alternative $B(x_2, p_2)$ using logistic regressions based on the: i) *EU* and *CPT* subjective utilities functions, and ii) relative number of fixations (or dwell-times) on each alternative. The *EU* based models which we examined were:

$$Traditional\ EU: P(x_1, p_1; x_2, p_2) = f(x_1^{\alpha} \cdot p_1 - x_2^{\alpha} \cdot p_2) \quad (1)$$

$$EU_{Dwell\ time}: P(x_1, p_1; x_2, p_2) = f(x_1^{\alpha} \cdot p_1 \cdot t_1^{\tau} - x_2^{\alpha} \cdot p_2 \cdot t_2^{\tau}) \quad (2)$$

$$EU_{Fixations}: P(x_1, p_1; x_2, p_2) = f(x_1^{\alpha} \cdot p_1 \cdot f_1^{\tau} - x_2^{\alpha} \cdot p_2 \cdot f_2^{\tau}) \quad (3)$$

where α is the risk-parameter of *EU*, t_1 and t_2 correspond to the relative looking time on the two alternatives (i.e., normalized by the total looking time), f_1 and f_2 correspond to the relative number of fixations (i.e., normalized by the total number of fixations) on the two alternatives, τ is a saturation parameter for fixations, and $f(x)$ is the logistic function, which depends on a slope-parameter, θ:

$$f(x) = \frac{1}{1+e^{-\theta \cdot x}}\ .$$

The *Traditional EU* model (Eq. 1) corresponds to a probabilistic specification of the *EU*, using an exponential version of Luce's choice rule [6,7], which takes into account the *EU* differences between the lotteries (see S3 Text for detailed description of the *EU* model). The $EU_{Dwell\ time}$ model (Eq. 2) includes the dwell-times on the two alternatives, so that the *EU* value of each alternative increases with its dwell time. The $EU_{Fixations}$ model (Eq. 3) is similar to the $EU_{Dwell\ time}$ model, except that instead of using dwell-time, we use the number of fixations to each alternative. Note that in both the latter models, we included the parameter τ, which represents the marginally decreasing effect of the dwell-time/number of fixations (lower values of this parameter indicate higher degrees of saturation; for example, if $\tau=0.5$, an increase of the fixation number by a factor of four will only increase the utility by a factor of two).

The *CPT* based models were analogues to the *EU* ones, except for using subjective probabilities (decision weights) rather than objective probabilities (as in *EU*):

$$Traditional\ CPT: P(x_1, p_1; x_2, p_2) = f(x_1^{\alpha} \cdot \pi(p_1) - x_2^{\alpha} \cdot \pi(p_2)) \quad (4)$$

$$CPT_{Dwell\ time}: P(x_1, p_1; x_2, p_2) = f(x_1^{\alpha} \cdot \pi(p_1) \cdot t_1^{\tau} - x_2^{\alpha} \cdot \pi(p_2) \cdot t_2^{\tau}) \quad (5)$$

$$CPT_{Fixations}: P(x_1, p_1; x_2, p_2) = f(x_1^{\alpha} \cdot \pi(p_1) \cdot f_1^{\tau} - x_2^{\alpha} \cdot \pi(p_2) \cdot f_2^{\tau}) \quad (6)$$

where $\pi(p) = \frac{p^{\gamma}}{(p^{\gamma}+(1-p)^{\gamma})^{\frac{1}{\gamma}}}$ is the decision weight function, and γ is a free parameter capturing the curvature of this function (see S3 Text for further details).

The quantitative fits of the models were evaluated using two selection criteria: prediction-accuracy and *AIC* (see S1 Methods for detailed description of each measure). The results indicate that: i) using eye-movements improve the prediction accuracy and *AIC* compared with the traditional models (Table S2), and ii) the fixations based models showed equal (for *EU*) or better (for *CPT*) performance than the dwell-time based models (Table S2).

Impact of overall fixations in EU and CPT type models

Model	***AIC***	***Prediction-Accuracy***
Traditional EU	2617	76.5%
$EU_{Dwell\ time}$	2449	80.5%
$EU_{Fixations}$	2447	79.9%
Traditional CPT	2364	81.2%
$CPT_{Dwell\ time}$	2248	83.5%
$\mathbf{CPT_{Fixations}}$	**2204**	**83.9%**

AIC values are rounded to the nearest integers. Bold entry indicates the best fitting models. Note that AIC differences exceeding 10 are considered very strong evidence in favor of the model with the lower numerical values.
