# Supplementary material for: The formation of preference in risky choice
Source: PLoS Comput Biol. 2019 Aug 29;15(8):e1007201. doi: 10.1371/journal.pcbi.1007201 (PMC6738658; doi:10.1371/journal.pcbi.1007201)
Supplement: S3 Text — (PDF) [file pcbi.1007201.s007.pdf]

S3 Text. Models of risky choice

Traditional Models

Expected Value (EV). The *EV* model assumes that the participants choose the alternative with the higher *EV* value. Because we used simple lotteries in the form of: (*x* ILS with a probability of *p*; otherwise nothing), the *EV* of each lottery in our study was defined as:

$$EV = x \cdot p$$

The probability of choosing each alternative is calculated using an exponential version of Luce's choice rule [6,7]:

$$P(x_1, p_1; x_2, p_2) = \frac{1}{1 + e^{-\beta(EV_A - EV_B)}}$$

where β indicates the sensitivity of the model to the differences between the *EV* of alternative A (EV_A) and the *EV* of alternative B (EV_B).

Expected Utility (EU). The *EU* model assumes that the participants choose the alternative with the higher expected utility, which was defined as:

$$EU = u(x) \cdot p$$

where $u(x) = x^\alpha$ is the utility function, and $\alpha \in [0,1]$ captures the curvature of this function (i.e., the risk-bias). As in the *EV* model, the probability of choosing each alternative was calculated using an exponential version of Luce's choice rule:

$$P(x_1, p_1; x_2, p_2) = \frac{1}{1 + e^{-\beta(EU_A - EU_B)}}$$

Cumulative Prospect Theory (CPT). According to *CPT* [1] the subjective utility of each lottery is given by:

$$SU_{CPT} = u(x) \cdot \pi(p)$$

where $u(x) = x^\alpha$ is the utility function (as in *EU*), and the decision weighting function is defined as follows:

$$\pi(p) = w\left(\sum_{i=j}^{n} p_i\right) - w\left(\sum_{i=j+1}^{n} p_i\right), 0 \le x_1 \le \cdots \le x_j \le \cdots \le x_n$$

$$w(p) = \frac{p^{\gamma}}{(p^{\gamma} + (1-p)^{\gamma})^{\frac{1}{\gamma}}}$$

where $w(p)$ is an *S*-shaped weighting function over-estimating low probabilities and under-estimating medium and high probabilities, and γ is a free parameter in [0,1] that captures the curvature of this function. Because we used simple lotteries in the form of A: (x_1 with p_1; otherwise 0) vs. B (x_2 with p_2; otherwise 0), the above equations can be reduced to:

$$\pi(p) = \frac{p^{\gamma}}{(p^{\gamma} + (1-p)^{\gamma})^{\frac{1}{\gamma}}}$$

We assumed that, as in the *EV* and *EU* models, the probability of choosing each alternative follows Luce's choice rule:

$$P(x_1, p_1; x_2, p_2) = \frac{1}{1 + e^{-\beta\left(SU_{CPT_A} - SU_{CPT_B}\right)}}$$

Heuristics

MaxiMax. The participants choose the alternative with the highest maximum outcome [8]. For example, when deciding between A:($20, 0.2; $0, 0.8) and B:($10, 0.5; $0, 0.5), according to the *MaxiMax* heuristic, alternative A (maximum outcome of $20) should be preferred over alternative B (maximum outcome of $10).

Least-Likely. The participants choose the alternative with the lowest probability of its worst outcome [9]. For example, when deciding between A:($20, 0.2; $0, 0.8) and B:($10, 0.5; $0, 0.5), according to the *Least-Likely* heuristic, alternative B (worst outcome probability of 0.5) should be preferred over alternative A (worst outcome probability of 0.8). Note that in our design the predictions of the *Least-Likely* heuristic were identical to those of the *MiniMax* heuristic (preference for the alternative with the higher minimum outcome [8]).

Priority Heuristic (PH). According to *PH*, the participants follow an ordered set of decision rules: first, the minimum outcomes of the alternatives are compared. In case the minimum outcomes differ by .1 (or more) of the maximum outcome, the alternative with the higher minimum outcome is chosen, otherwise the probabilities of the minimum outcomes are compared. In case these probabilities differ by .1 (or more), the alternative with the lower probability (of the minimum outcome) is chosen,

otherwise the alternative with the higher probability of its maximum outcome is chosen. For example, when deciding between A:($20, 0.2; $0, 0.8) and B:($10, 0.5; $0, 0.5), according to *PH*, alternative B should be preferred over alternative A. This is because the minimum outcomes of these alternatives are identical, however the probability of the minimum outcome of alternative A ($p_{minimum\ outcome}$ = 0.8) is higher than that of alternative B ($p_{minimum\ outcome}$ = 0.5).

Within-attribute Selection

Normalized differences. The *within-attribute selection: normalized differences* model consisted of two leaky accumulators (Y_A and Y_B) integrating the normalized differences of the attended attribute (amount and probability) at each step according to the following difference equations:

$$Y_A(t+1) = (1-\lambda)\cdot Y_A(t) + D_A(t)$$
$$Y_B(t+1) = (1-\lambda)\cdot Y_B(t) + D_B(t)$$

where t denotes the number of fixations from the beginning of the trial, and λ is a free parameter representing the integration leak.

The two accumulators were initialized at 0:

$$Y_A(0) = Y_B(0) = 0$$

In case of fixations toward the amount of alternative A (x_1) the inputs to the accumulators ($D_{A,B}(t)$), were defined as follows:

$$D_A(t) = x_1{}' - \theta\cdot x_2{}'$$
$$D_B(t) = \theta\cdot x_2{}' - x_1{}'$$

and in case of fixations to the amount of alternative B (x_2), $D_{A,B}(t)$ were defined as:

$$D_A(t) = \theta\cdot x_1{}' - x_2{}'$$
$$D_B(t) = x_2{}' - \theta\cdot x_1{}'$$

Similarly, in case of fixations toward the probability of alternative A (p_1) the inputs were defined as:

$$D_A(t) = p_1{}' - \theta\cdot p_2{}'$$
$$D_B(t) = \theta\cdot p_2{}' - p_1{}'$$

and in case of fixations to the probability of alternative B (p_2), they were defined as:

$$D_A(t) = \theta\cdot p_1{}' - p_2{}'$$
$$D_B(t) = p_2{}' - \theta\cdot p_1{}'$$

Where the prime symbol denotes rescaling of the amounts and probabilities by applying the min-max normalization, using the following formula:

$$y' = \frac{y - min(y)}{max(y) - min(y)}$$

where min(y) and max(y) denotes the minimum and maximum values (amounts or probabilities) in the set.

The parameter $\theta \in [0,1]$, represents attentional modulation (i.e., the reduction of the accumulation gain for the unattended alternative [3]).

This model assumes some degree of working memory (or peripheral vision) representation of the unattended attribute. Because working memory cannot play a role at the first fixation on an attribute (and since peripheral vision is less sensitive to the low contrast of our stimuli), we assumed that at first fixations a default value (mid-range) is used for the non-scanned attribute. For example, if a participant fixates on x_1, but has not yet fixated on x_2, a default value of 15 (mid-range value) would be assigned to this attribute when calculating $D_A(t)$ and $D_B(t)$. Similarly, a default value of 0.5 (mid-range values) would be assigned to yet non-scanned probabilities.

At the end of each trial, the model chooses alternative A, with a probability of:

$$p(A) = \frac{1}{1 + e^{-\beta(Y_A - Y_B)}}$$

and alternative B with a probability of:

$$p(B) = 1 - p(A)$$

where Y_A and Y_B are the accumulated values of alternative A and B at the end of the trial, and β is the sensitivity parameter.

Note that our approach differs from previous work with the *aDDM* (but see [5]), in that units of accumulation are the number of fixations rather than fixation time. For example, this means that a fixation of 200ms contributes the same as one of 400ms. There are a few reasons that motivated us to make this assumption, other than minimizing computational complexity. First, neural studies show that responses in high-visual areas (unlike in early ones) are invariant to the duration of the retinal

stimulation or the length of fixation [10]. Note also that in our experiment the fixations shift between four attributes, allowing a differential number of fixations to the alternatives (for example a sequence of transitions x_1, p_1, x_1 adds 3 fixations to alternative 1). Finally, we found that time-based accumulation did not improve the prediction accuracy compared with fixation-based ones. In fact, our best fitting model, (within-alternative/2-layer leaky accumulators), showed better performance when the units of accumulation were the number of fixations (AIC = 1877), rather than fixation time (AIC = 2154; see alsoS2 Text).

Categorical differences. This model was implemented as the *within-attribute selection: normalized differences* model, except for the following differences:

1. Amounts and probabilities were not rescaled using the min-max normalization.
2. The accumulators (Y_A and Y_B) did not integrate the normalized differences, but rather counts based on categorical comparisons (*DbS*; [11–13]). Thus, in a case of fixations toward the amount of alternative A (x_1), for example, the inputs to the accumulators ($D_{A,B}(t)$), were defined as follows:

$$D_A(t) = f(u(x_1), \theta \cdot u(x_2))$$
$$D_B(t) = f(\theta \cdot u(x_2), u(x_1))$$

where $f(x, y)$ is a step function defined as:

$$f(x,y) = \begin{cases} 1, if x > y \\ 0, if x < y \end{cases}$$

In case of fixations toward p_1, x_2 and p_2, the inputs to the accumulators were defined analogously.

Within alternative integration

One-layer leaky accumulators. The one-layer model consisted of two leaky accumulators (Y_A and Y_B) integrating the subjective utilities of the two lotteries (A and B) according to the following difference equations:

$$Y_A(t+1) = (1-\lambda) \cdot Y_A(t) + SU_A(t)$$
$$Y_B(t+1) = (1-\lambda) \cdot Y_B(t) + SU_B(t)$$

where t denotes the number of fixations from the beginning of the trial, and λ is a free parameter representing the integration leak.

The two accumulators are initialized at 0:

$$Y_A(0) = Y_B(0) = 0$$

In case of fixations toward alternative A the inputs to the accumulators $SU_{A,\,B}(t)$, are defined as follows:

$$SU_A(t) = u(x_A) \cdot \pi(p_A)$$
$$SU_B(t) = \theta \cdot u(x_B) \cdot \pi(p_B)$$

and in case of fixations toward alternative B the inputs are defined as follows:

$$SU_A(t) = \theta \cdot u(x_A) \cdot \pi(p_A)$$
$$SU_B(t) = u(x_B) \cdot \pi(p_B)$$

where θ is a free parameter represent attentional modulation, $u(x) = x^{\alpha}$ is the utility function (as in the implementations of *EU* and *CPT*), $\pi(p) = \frac{p^{\gamma}}{p^{\gamma}+(1-p)^{\gamma}}$ is the decision weight function similar to the one implemented in the *CPT*, except for the denominator is not raised to the power of $1/\gamma$ as in the *CPT* model [1]. This can be interpreted as a psychophysical transformation of probabilities judgment [14].

Here, as in the *within-attribute selection* models, we assume that mid-range default values of 15 (amount) and 0.5 (probabilities) are assigned to yet non-scanned attributes (see *Within-attribute Selection: Normalized differences* for more details).

At the end of each trial, the model chooses alternative A, with a probability of:

$$p(A) = \frac{1}{1 + e^{-\beta(Y_A - Y_B)}}$$

and alternative B with a probability of:

$$p(B) = 1 - p(A)$$

where Y_A and Y_B are the accumulated values of alternative A and B at the end of the trial, and β is the sensitivity parameter.

Two-layer leaky accumulators. The model contains two layers of leaky-accumulators in cascade. The first layer consists of four leaky-accumulators *(X_1, P_1, X_2, P_2)*, representing the activations of the different attributes (x_1, p_1, x_2, p_2). These accumulators are updated according to the following difference equations:

$$X_1(t+1) = (1-\lambda) \cdot X_1(t) + w_{x_1}(gaze\ location) \cdot u(x_1)$$
$$P_1(t+1) = (1-\lambda) \cdot P_1(t) + w_{p_1}(gaze\ location) \cdot \pi(p_1)$$

$$X_2(t+1) = (1-\lambda)\cdot X_2(t) + w_{x_2}(gaze\ location)\cdot u(x_2)$$

$$P_2(t+1) = (1-\lambda)\cdot P_2(t) + w_{p_2}(gaze\ location)\cdot \pi(p_2)$$

where *t* denotes the number of fixations from the beginning of the trial, λ represents the integration leak, $u(x) = x^{\alpha}$ is the utility function, $\pi(p) = \frac{p^{\gamma}}{p^{\gamma}+(1-p)^{\gamma}}$ is the decision weight function (see *CPT* and *within-attribute selection: normalized differences* sections for further details). w_{x_1} is a step function defined as:

$$w_{x_1}\,(gaze\ location) = \begin{cases} 1, if gaze\ is\ directed\ to\ x_1 \\ \theta, otherwise \end{cases}$$

where θ in [0,1], is a free parameter representing attentional modulation w_{p_1}, w_{x_2} and w_{p_2} are defined analogously to w_{x_1}.

The second layer consisted of two leaky accumulators (Y_A and Y_B) integrating the subjective utilities of the two lotteries (*A* and *B*) according to the following difference equations:

$$Y_A(t+1) = (1-\lambda)\cdot Y_A(t) + X_1(t)\cdot P_1(t)$$

$$Y_B(t+1) = (1-\lambda)\cdot Y_B(t) + X_2(t)\cdot P_2(t)$$

where *t* denotes the number of fixations from the beginning of the trial, λ is a free parameter representing the integration leak, and $X_1(t), P_1(t), X_2(t), P_2(t)$ represent the activations of the different attributes at time t.

All accumulators are initialized at 0:

$$X_1(0) = P_1(0) = X_2(0) = P_2(0) = 0$$

$$Y_A(0) = Y_B(0) = 0$$

Here, as in the *within-attribute selection* models, we assume that mid-range default values of 15 (amount) and 0.5 (probabilities) are assigned to yet non-scanned attributes (see *Within-attribute Selection: Normalized differences* for more details).

At the end of each trial, the model chooses alternative A, with a probability of:

$$p(A) = \frac{1}{1+e^{-\beta(Y_A - Y_B)}}$$

and alternative B with a probability of:

$$p(B) = 1 - p(A)$$

where Y_A and Y_B are the accumulated values of alternative A and B at the end of the trial, and β is the noise parameter.

We also tested a more hybrid version of this model that includes mutual inhibition between the amount units (X_1 and X_2) and the probability units (P_1 and P_2). In this version, the first layer leaky-accumulators are updated according to the following difference equations:

$$X_1(t+1) = (1-\lambda)\cdot X_1(t) + w_{x_1}(gaze\ location)\cdot u(x_1) - \omega_x \cdot X_2(t)$$

$$P_1(t+1) = (1-\lambda)\cdot P_1(t) + w_{p_1}(gaze\ location)\cdot \pi(p_1) - \omega_p \cdot P_2(t)$$

$$X_2(t+1) = (1-\lambda)\cdot X_2(t) + w_{x_2}(gaze\ location)\cdot u(x_2) - \omega_x \cdot X_1(t)$$

$$P_2(t+1) = (1-\lambda)\cdot P_2(t) + w_{p_2}(gaze\ location)\cdot \pi(p_2) - \omega_p \cdot P_1(t)$$

where ω_x is the mutual inhibition between X_1 and X_2 and ω_p is the mutual inhibition between P_1 and P_2. As this model has two extra parameters (ω_x and ω_p), we kept two of the other parameters (leak and attentional modulation) to the optimal values of the model without mutual inhibition. We wish to point out, however, that inhibition at the level of attributes is not motivated by Connectionist principles [15], which suggested mutual inhibition between units that correspond to different alternatives (for example, this could apply to inhibition between alternative A and B at the 2nd alternative layer, see Fig. 6A).
