# Supplementary material for: The formation of preference in risky choice
Source: PLoS Comput Biol. 2019 Aug 29;15(8):e1007201. doi: 10.1371/journal.pcbi.1007201 (PMC6738658; doi:10.1371/journal.pcbi.1007201)
Supplement: S4 Text — (PDF) [file pcbi.1007201.s008.pdf]

S4 Text. Transitivity Violations

The axiom of transitivity is one of the cornerstones of normative decision theories. Lack of transitivity implies a non-consistent decision making process, in which stochastic fluctuations occur in the way individuals retrieve relevant information, weight the different aspects of the alternatives or integrate these weights in order to reach a decision [25,26]. To estimate the degree of intransitivity of the different participants, we computed for each participant the fraction of triples in which alternative x was preferred over alternative y, alternative y was preferred over alternative z, but alternative z was preferred over alternative x.
