# Supplementary material for: The formation of preference in risky choice
Source: PLoS Comput Biol. 2019 Aug 29;15(8):e1007201. doi: 10.1371/journal.pcbi.1007201 (PMC6738658; doi:10.1371/journal.pcbi.1007201)
Supplement: S1 Methods — (PDF) [file pcbi.1007201.s009.pdf]

S1 Methods. Supplementary experimental methods.

Optimization procedure (choices). The free parameters of the different risky choice models were fitted to the data of each participant separately, using maximum likelihood estimation. We first constructed an n-dimensional grid (n is the number of free parameters for each model), with α, γ, λ and θ values ranging from 0 to 1 in increments of 0.25, and β values ranging from 0 to 10 in increments of 1. This grid was searched exhaustively, and for each set of parameters, θ_j, the likelihood was calculated according to the following formula:

$$L(\theta_j) = \prod_{i=1}^{N} p_i^{R_i} \cdot (1 - p_i)^{1-R_i}$$

where R_i is a dichotomous variable that equal to one if the participants chose the upper alternative and to zero otherwise, and p_i denotes the probability of this response predicted by the model. The 10 parameters sets that had the highest likelihood were fed as starting points to a Simplex minimization routine [16], in which the cost function was defined as the negative log-likelihood. The mean best-fitting parameters (averaged across participants) are shown in Table S3.

Optimization procedure (choices and decision-times). The optimization procedure for both choices and decision-times was performed in two steps: i) We fitted the models only to choices (using the procedure described in the previous section), and ii) We deployed an integration-to-boundary framework [17,18] and fitted the models simultaneously to choices and decision-times (based on the number of fixations), using the best fitted parameters obtained in the first step, together with a new noise and boundary parameters. We tested two types of boundaries: i) fixed boundaries:

$$u(t) = a$$

$$l(t) = -u(t)$$

where $u(t)$ and $l(t)$ are the upper and lower thresholds at time t, and a is a constant. and ii) collapsing boundaries, modeled using a Weibull cumulative distribution function [19,20]:

$$u(t) = a - \left(1 - exp\left(-\left(\frac{t}{\lambda}\right)^k\right)\right) \cdot (a - a')$$

$$l(t) = -u(t)$$

where $u(t)$ and $l(t)$ are the upper and lower thresholds at time t, respectively, a is the initial value of the boundary, a' is the asymptotic value of the boundary, λ and k are the scale and shape parameters of the Weibull function, respectively.

We used maximum likelihood estimation (MLE) to fit the models to each subject data. For each trial, we simulated the model 1,000 times for a given set of proposal parameters and calculated the proportion of trials in which the model choice and decision-time (in number of fixations) matched the empirical data. Denoting this proportion by p_i, we maximized the likelihood function $L(D|\theta)$ of the data (D) given a set of proposal parameters (θ), by:

$$L(D|\theta) = \prod_{i=1}^{N} p_i$$

To find the best set of proposal parameters we first used an adaptive grid search algorithm (see [21] for details) and then used the best set of proposal parameters as starting points to a Simplex minimization routine [16].

Model selection. In order to evaluate the quantitative fits of the models, we used several methods: i) prediction accuracy, ii) Akaike Information Criterion (*AIC*; [22]), and iii) cross-validation. To operationalize the first selection criterion, we calculated the percentage of match between the choices of each participant and the deterministic predictions of the different models (derived using the best fitting set of parameters for each model). The *AIC* implement a trade-off between model goodness of fit and complexity by penalizing additional free parameters according to the following formulas:

$$AIC = -2 \cdot LL + 2 \cdot k$$

where *LL* is the log-likelihood for the best fitting parameters, *k* is the number of free parameters. *AIC* differences exceeding 10 are considered decisive evidence in favor of the model with the lower numerical values [23].

Additionally, we also employed 5-fold cross-validation to evaluate predictive validity [24]. In this method, the data is randomly partitioned into five non-overlapping subsamples. Four out of them are then used to estimate the maximum likelihood parameters. These parameters are then fixed and used to compute the G^2 statistic ($-2 \cdot LL$) and the *accuracy* in the remaining subsample. This process is repeated five times, with each subsample serving only once as the validation data.
