# Supplementary figures and images for: The formation of preference in risky choice
Source: PLoS Comput Biol. 2019 Aug 29;15(8):e1007201. doi: 10.1371/journal.pcbi.1007201 (PMC6738658; doi:10.1371/journal.pcbi.1007201)

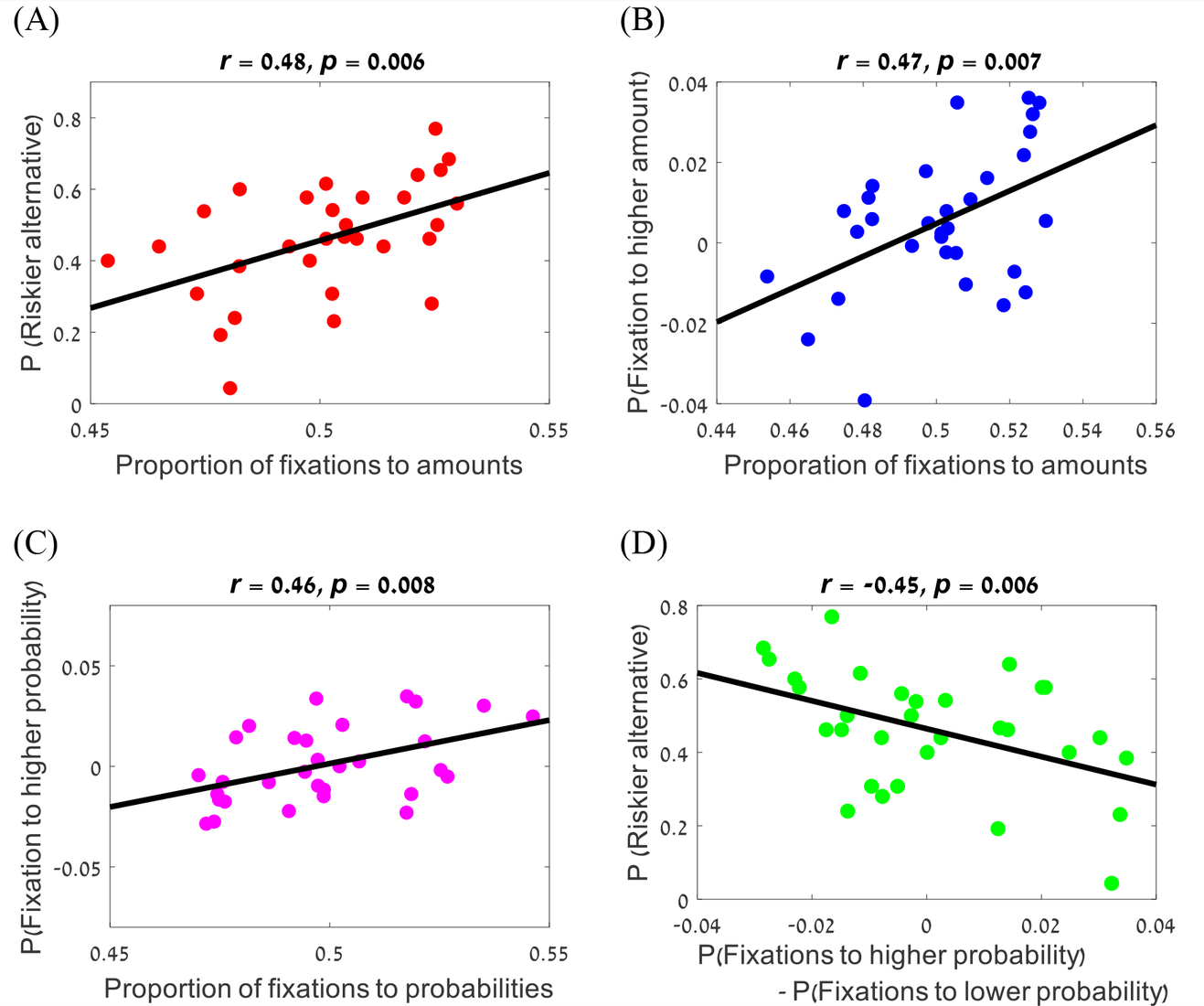

Supplement: S1 Fig — Correlations between the proportion of fixations toward amounts/probabilities and risk-attitudes. (A) The proportion of fixations toward amounts was positively correlated with preference for the riskier alternative. (B) The proportion of fixations toward amounts was positively correlated with the tendency to fixate on the larger amount. (C) The proportion of fixations toward probabilities was positively correlated with the tendency to fixate on the larger probability. (D) The difference in proportion of fixations to higher and lower probabilities was negatively correlated with risk-seeking preference. (TIF) [file pcbi.1007201.s010.tif]

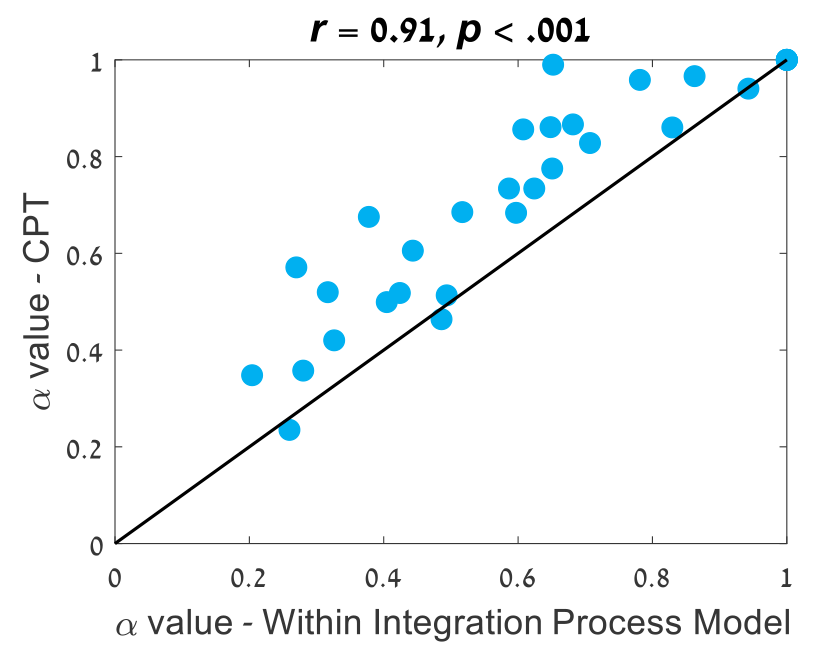

Supplement: S2 Fig — The utility curvature parameters (α) of our process model and of the CPT model were highly correlated across participants. However, the CPT parameters have higher values (the solid black line corresponds to the identity line). This may suggest that the CPT parameters reflect a combination of different components, such as selective allocation of attention and subjective value transformation [65]. (TIF) [file pcbi.1007201.s011.tif]

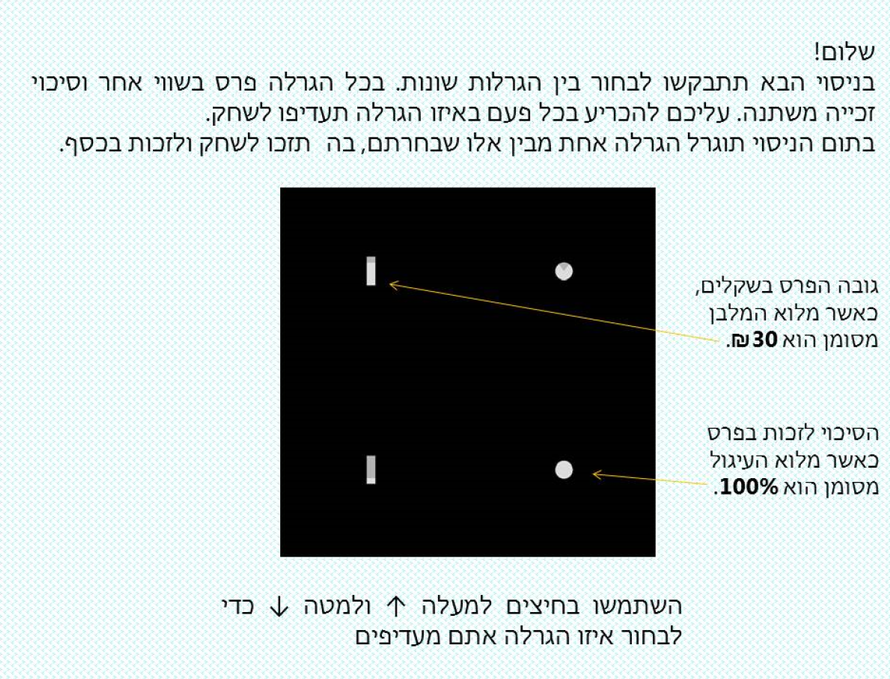

Supplement: S3 Fig — At the beginning of the experiment the above instructions were presented to the participants. The experimenter read them out loud and verified that the participants fully understand the task. The text translates from Hebrew as follows: "Hello! In the following experiment you will be asked to choose between different lotteries. Each lottery holds a different amount of prize money and different chances of winning. You will be asked to choose which of two lotteries you prefer. At the end of the experiment, one of the lotteries would be randomly chosen, and you will get the chance to play and win real money. Yellow arrow towards the upper bar: the amount of money when the bar is full is 30 Israeli shekels. Yellow arrow towards the lower pie chart: the chance of winning the prize when the entire pie is full is 100%. Use the ↑ and ↓ arrows to choose which lottery you prefer". (TIF) [file pcbi.1007201.s012.tif]

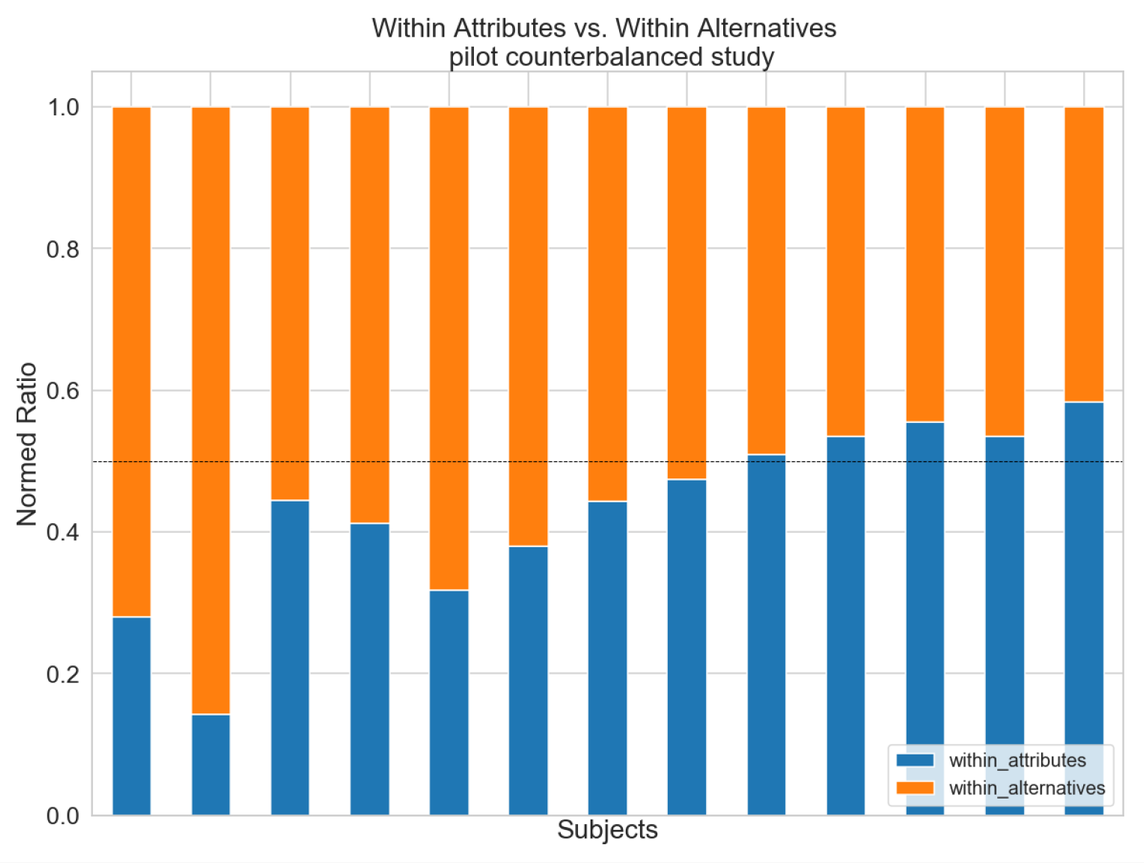

Supplement: S4 Fig — In a pilot study conducted before the main experiment, the lotteries were displayed side by side (i.e., horizontal alignment) with the bars presented always on top and the pie-charts presented always underneath. In this study, the participants made more within-alternative than within-attribute transitions (as in the experiment reported in the main text). (TIF) [file pcbi.1007201.s013.tif]

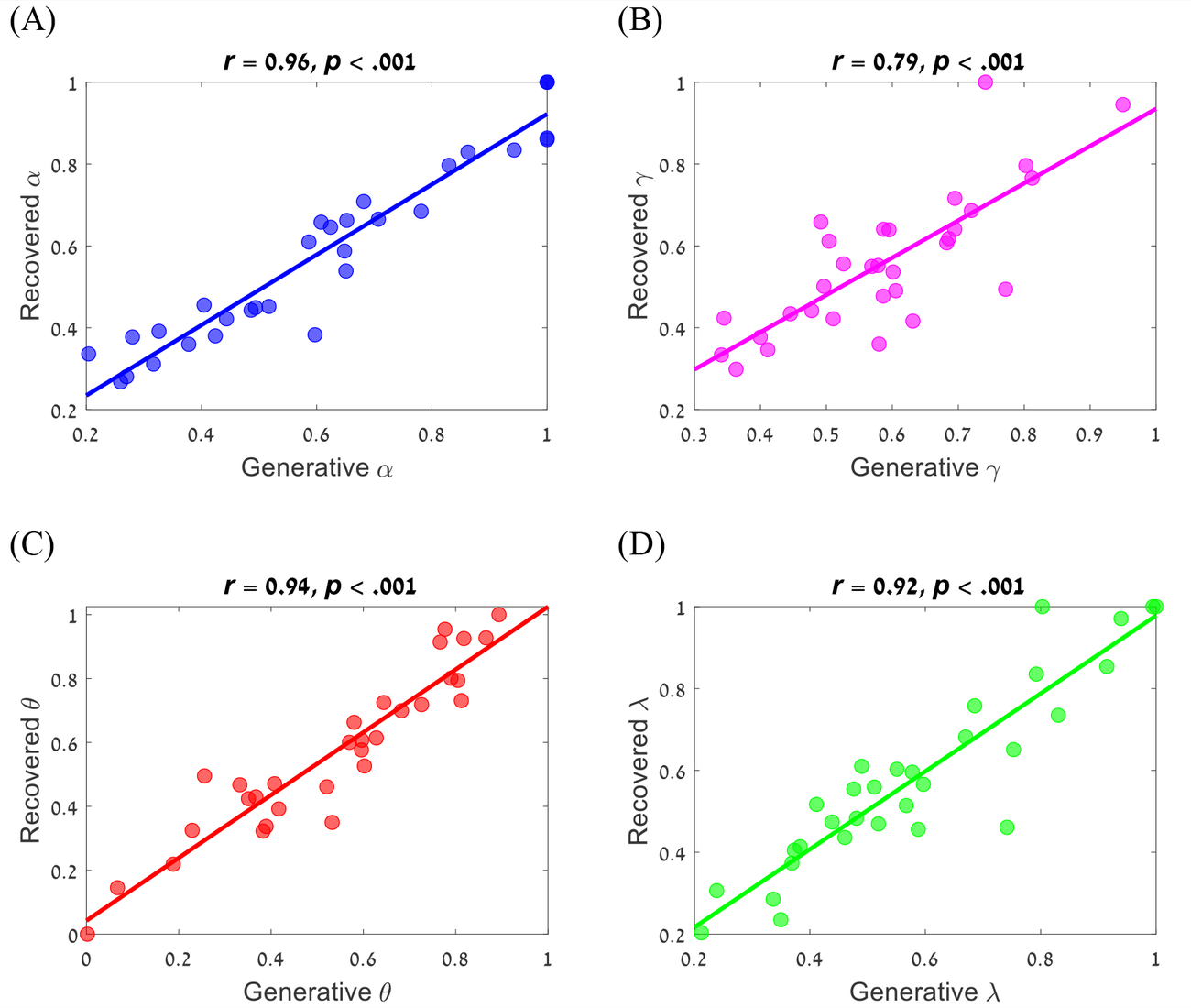

Supplement: S5 Fig — The ability of the fitting procedure to accurately identify the parameters of our best-fitting model (within-alternative/two-layer leaky accumulators) was tested by simulating the model using the participants’ estimated parameters. For each parameter, we simulated the model for the 94 non-dominated experimental trials. The figure shows the generative parameters of the model plotted against the recovered parameters, for (A) α-risk aversion (B) γ-probability weighting (C) θ-attentional modulation and (D) λ-activation leak for simulated data. (TIF) [file pcbi.1007201.s014.tif]
