# Supplementary material for: The formation of preference in risky choice
Source: PLoS Comput Biol. 2019 Aug 29;15(8):e1007201. doi: 10.1371/journal.pcbi.1007201 (PMC6738658; doi:10.1371/journal.pcbi.1007201)
Supplement: S1 References — (PDF) [file pcbi.1007201.s015.pdf]

S1 References.

1. Tversky A, Kahneman D. Advances in prospect theory: Cumulative representation of uncertainty. J Risk Uncertain. 1992;5:297-323. doi:10.1007/BF00122574

2. Kahneman D, Tversky A. Prospect Theory: An Analysis of Decision under Risk. Econometrica. 1979;47:263-291. doi:10.2307/1914185

3. Krajbich I, Armel C, Rangel A. Visual fixations and the computation and comparison of value in simple choice. Nat Neurosci. 2010;13:1292-1298. doi:10.1038/nn.2635

4. Shimojo S, Simion C, Shimojo E, Scheier C. Gaze bias both reflects and influences preference. Nat Neurosci. 2003;6:1317-1322. doi:10.1038/nn1150

5. Stewart N, Hermens F, Matthews WJ. Eye movements in risky choice. J Behav Decis Mak. 2016;29:116-136. doi:10.1002/bdm.1854

6. Luce RD. Individual Choice Behavior: A Theoretical Analysis. Oxford: Wiley; 1959.

7. Rieskamp J. The probabilistic nature of preferential choice. J Exp Psychol Learn Mem Cogn. 2008;34:1446.

8. Coombs CH, Dawes RM, Tversky A. Mathematical Psychology: An Elementary Introduction. Prentice-Hall; 1970.

9. Thorngate W. Efficient decision heuristics. Syst Res Behav Sci. 1980;25:219-225. doi:10.1002/bs.3830250306

10. Podvalny E, Yeagle E, Mégevand P, et al. Invariant temporal dynamics underlie perceptual stability in human visual cortex. Curr Biol. 2017;27:155-165. doi:10.1016/j.cub.2016.11.024

11. Stewart N. Decision by sampling: The role of the decision environment in risky choice. Q J Exp Psychol. 2009;62:1041-1062. doi:10.1080/17470210902747112

12. Stewart N, Chater N, Brown GDA. Decision by sampling. Cogn Psychol. 2006;53:1-26. doi:10.1016/j.cogpsych.2005.10.003

13. Stewart N, Simpson K. A decision-by-sampling account of decision under risk. probabilistic mind Prospect Bayesian Cogn Sci. 2008:261-276. doi:10.1093/acprof:oso/9780199216093.003.0012

14. Hollands JG, Dyre BP. Bias in proportion judgments: The cyclical power model. Psychol Rev. 2000;107:500-524. doi:10.1037/0033-295X.107.3.500

15. McClelland JL. Toward a theory of information processing in graded, random, and interactive networks. 1993.

16. Nelder JA, Mead R. A Simplex Method for Function Minimization. Comput J. 1965;7:308-313. doi:10.1093/comjnl/7.4.308

17. Ratcliff R, McKoon G. The diffusion decision model: theory and data for two-choice decision tasks. Neural Comput. 2008;20:873-922. doi:10.1162/neco.2008.12-06-420

18. Ratcliff R, Smith PL, Brown SD, McKoon G. Diffusion decision model: current issues and history. Trends Cogn Sci. 2016;20:260-281. doi:10.1016/j.tics.2016.01.007

19. Hawkins GE, Forstmann BU, Wagenmakers E-J, Ratcliff R, Brown SD. Revisiting the Evidence for Collapsing Boundaries and Urgency Signals in Perceptual Decision-Making. J Neurosci. 2015;35:2476-2484. doi:10.1523/jneurosci.2410-14.2015

20. Palestro JJ, Weichart E, Sederberg PB, Turner BM. Some task demands induce collapsing bounds Evidence from a behavioral analysis. Psychon Bull Rev. 2018. doi:10.3758s13423-018-1479-9

21. Tavares G, Perona P, Rangel A. The Attentional Drift Diffusion Model of Simple Perceptual Decision-Making. Front Neurosci. 2017;11:468. doi:10.3389/fnins.2017.00468

22. Akaike H. A new look at the statistical model identification. IEEE Trans

Automat Contr. 1974;19:716-723. doi:10.1109/TAC.1974.1100705

23. Burnham KP, Anderson DR. Model selection and multimodel inference New York. NY Springer. 2002.

24. Browne MW. Cross-Validation Methods. J Math Psychol. 2000;44:108-132. doi:10.1006/jmps.1999.1279

25. Lee L, Amir O, Ariely D. In Search of Homo Economicus: Cognitive Noise and the Role of Emotion in Preference Consistency. J Consum Res. 2009;36:173-187. doi:10.1086/597160

26. Tversky A. Intransitivity of preferences. Psychol Rev. 1969;76:31-48. doi:10.1037/h0026750

27. Pachur T, Schulte-Mecklenbeck M, Murphy RO, Hertwig R. Prospect theory reflects selective allocation of attention. J Exp Psychol Gen. 2018;147:147-169. doi:10.1037/xge0000406
